# Bacterial Drug Tolerance under Clinical Conditions Is Governed by Anaerobic Adaptation but not Anaerobic Respiration

**DOI:** 10.1128/AAC.02793-14

**Published:** 2014-10

**Authors:** Claudia M. Hemsley, Jamie X. Luo, Clio A. Andreae, Clive S. Butler, Orkun S. Soyer, Richard W. Titball

**Affiliations:** a Department of Biosciences, College of Life and Environmental Sciences, University of Exeter, Exeter, United Kingdom; b School of Life Sciences, University of Warwick, Coventry, United Kingdom

## Abstract

Noninherited antibiotic resistance is a phenomenon whereby a subpopulation of genetically identical bacteria displays phenotypic tolerance to antibiotics. We show here that compared to Escherichia coli, the clinically relevant genus Burkholderia displays much higher levels of cells that tolerate ceftazidime. By measuring the dynamics of the formation of drug-tolerant cells under conditions that mimic *in vivo* infections, we show that in Burkholderia bacteria, oxygen levels affect the formation of these cells. The drug-tolerant cells are characterized by an anaerobic metabolic signature and can be eliminated by oxygenating the system or adding nitrate. The transcriptome profile suggests that these cells are not dormant persister cells and are likely to be drug tolerant as a consequence of the upregulation of anaerobic nitrate respiration, efflux pumps, β-lactamases, and stress response proteins. These findings have important implications for the treatment of chronic bacterial infections and the methodologies and conditions that are used to study drug-tolerant and persister cells *in vitro*.

## INTRODUCTION

In a genetically identical population of bacteria, high doses of antibiotics kill most but not all individuals. On reculturing, the surviving bacteria give rise to a population that is similarly susceptible to antibiotics. This phenomenon was first identified almost 70 years ago (1) and has been termed noninherited antibiotic resistance. The molecular mechanisms that enable cells to resist supra-MIC doses of antibiotics are not fully elucidated. In some cases, drug tolerance is a consequence of the formation of dormant persister cells (2, 3). There is now evidence linking bacterial drug tolerance and persister cell formation with chronic infections, which are refractory to treatment with antibiotics (4–6).

Many different explanatory hypotheses explaining bacterial drug tolerance and persister cell formation have been proposed (3, 7, 8). One prominent idea is that there are two phenotypically distinct cell states between which bacteria switch at constant rates (9, 10). However, this and other hypotheses are derived mainly from experiments with Escherichia coli grown in aerated broth cultures. This does not reflect the conditions under which bacteria that establish chronic disease encounter *in vivo* during antibiotic treatment. Chronic infections are often characterized by the formation of biofilms (11, 12) or the existence of bacteria in structures, such as granulomas. For example, Mycobacterium tuberculosis, the causative agent of tuberculosis, forms granulomas in the lungs (13), and Burkholderia pseudomallei, the causative agent of melioidosis, forms granulomas and abscesses in various organs of infected mice and humans (14–16). In these structured environments, bacteria are subjected to nutrient-starved low-oxygen conditions, and studies in several bacterial species have shown that their metabolic state is more similar to that of stationary-phase bacteria than to that of log-phase bacteria grown in the laboratory (17, 18).

In this study, we investigated the formation of drug-tolerant cells by B. pseudomallei and its close but nonpathogenic relative Burkholderia thailandensis. Both bacteria can be readily isolated from soil or stagnant water in many tropical countries and can persist in the environment. For example, B. pseudomallei is reported to survive for extended periods in moist clay soil, in water at different pH or salt concentrations, or even in nutrient-free water for up to 16 years (19–22). B. pseudomallei and B. thailandensis share highly syntenic chromosomes (23) and form a monophyletic clade (24), and B. thailandensis is frequently used as a surrogate for B. pseudomallei.

Here, we show that B. pseudomallei and B. thailandensis form drug-tolerant cells at a high frequency. Using transcriptional profiling, *in vivo*-like experimental conditions, and mathematical modeling, we demonstrate that the formation of these drug-tolerant cells is affected by oxygen and nitrate and that these cells adopt an anaerobic metabolism. In accordance with this model, we show that increasing oxygen levels results in a decrease in the number of drug-tolerant cells *in vitro*. These findings provide important new insights into the mechanisms of formation of drug-tolerant cells during disease.

## MATERIALS AND METHODS

### Bacterial strains and culture conditions.

All bacterial strains are listed in Table S1 in the supplemental material. B. thailandensis strain E264 was mainly used throughout the study. Unless stated otherwise, the bacteria were grown with aeration at 200 rpm in Luria-Bertani broth (LB) at 37°C. Anaerobic incubation was performed in an anaerobic chamber (DG 500 workstation; Don Whitley Scientific). Ceftazidime (catalog no. C3809; Sigma-Aldrich) was used at 400 μg/ml by preparing dilutions from a freshly prepared stock at 10 mg/ml active component in 0.1 N NaOH. Ciprofloxacin (catalog no. 17850; Sigma-Aldrich) was used at 40 μg/ml by preparing dilutions from a 1-mg/ml stock solution in 0.1 N NaOH, which was stored at −20°C. Metronidazole (catalog no. M1547; Sigma-Aldrich) was used at 100 μg/ml.

### Construction of a Δ*narG* mutant.

An in-frame deletion mutant was constructed using suicide plasmid-containing regions homologous to the regions upstream and downstream of the target genes (25). The amplified DNA fragments used for constructing the suicide plasmid derivatives were generated by recombinant PCR (26). Briefly, 600-bp upstream and downstream fragments of the *narG* (BPSL2309) gene of B. pseudomallei strain K96243 were PCR amplified using two different primer combinations: p2309-1 and p2309-2 (upstream), and p2309-3 and p2309-4 (downstream), with K96243 genomic DNA as a template. Both the p2309-2 and pD2309-3 primers contained complementary sequences, containing a HindIII restriction site and a start and stop codon of the target gene to allow for efficient ligation of both flanking regions of the target gene by fusion PCR. The resulting PCR product (1.2 kb) was cloned into the suicide vector pDM4 via its NdeI and XbaI sites and confirmed by PCR using the upstream (p2309-1) and downstream (p2309-4) primers. The pDM4 derivative was transformed into E. coli strain S17 λ*pir* and conjugated into B. pseudomallei by mating. Recombinant B. pseudomallei strains were selected on LB agar plates supplemented with 100 μg/ml gentamicin to select against the donor strain and 100 μg/ml chloramphenicol to select for B. pseudomallei transconjugants carrying the pDM4 constructs integrated into the chromosome. To generate in-frame deletion mutants, overnight cultures of the transconjugants were plated onto LB agar plates containing 10% (wt/vol) sucrose. Chloramphenicol-sensitive colonies were analyzed for a deletion of the target gene. Confirmation of the mutants was performed by two PCRs (see Fig. S7A in the supplemental material), using primers binding to an internal 300-bp region of the gene (absent in the mutant) and a second PCR using primers binding 300 bp upstream and downstream of the target gene (BPSL2309).

### Drug tolerance under hypoxic conditions.

The fraction of drug-tolerant cells in a population was determined by exposing the bacterial cultures to antibiotics using a concentration of >100× the MIC for ceftazidime (400 μg/ml for strain E264) or 10× the MIC for ciprofloxacin (40 μg/ml for strain E264), concentrations at which the antibiotics were still soluble. In order to mimic slow-growing hypoxic *in vivo* conditions, the assay was carried out using stationary-phase cultures, which were distributed into 24-well plates (nonpyrogenic polystyrene; Corning) using a standard volume of 1 ml per sample containing approximately 10^8^ CFU each. When necessary, 20 mM sodium nitrate was added to the appropriate wells of the 24-well plate. The assay plates were incubated statically at 37°C for 24 h in an aerobic incubator, unless stated otherwise. At the end of the incubation period, the samples were transferred to centrifuge tubes and centrifuged for 4 min at maximum speed. The antibiotic-containing supernatant was removed, and the cells were resuspended in 1 ml fresh LB medium. CFU counts were performed by spot plating serial dilutions onto LB agar plates. The survival frequency was defined as the number of cells that survived the antibiotic treatment divided by the input CFU. Killing curve analyses were performed in a similar manner by preparing 24 1-ml aliquots of bacteria in an antibiotic solution in a 24-well plate that was incubated statically at 37°C. One-milliliter samples were removed at the indicated time points and processed as described above.

### Isolation of bacterial mRNA.

Total RNA was extracted from 13 ml of exponentially growing cultures (optical density at 600 nm [OD_600_], 0.5 to 0.6), 4.5 ml of stationary-phase cultures at 16 h after inoculation (OD_600_, 4.5 to 5.5), and 24-ml pooled drug-tolerant cells (equal to the assay mixture after 24 h of treatment with the lytic antibiotic ceftazidime, which should mostly contain RNA from surviving cells), each in triplicate, using a hot phenol extraction protocol (27). In brief, the cells were harvested by centrifugation at 4,000 rpm at 4°C for 10 min. The cell pellet was resuspended in 1 ml of ice-cold resuspension buffer (10 mM KCl, 5 mM MgCl_2_, 10 mM Tris [pH 7.4]), and 0.5 ml each was added to preheated tubes containing 0.4 ml of lysis buffer (0.4 M NaCl, 40 mM EDTA, 1% β-mercaptoethanol, 1% sodium dodecyl sulfate, 20 mM Tris [pH 7.4]) and 0.2 ml of acid phenol (pH 4.5) (catalog no. AM9720; Ambion), using duplicate tubes per sample. The tubes were incubated at 90°C for 5 min and then chilled on ice for 5 min. Phase separation was achieved by centrifugation at 13,000 rpm for 2 min. RNA in the supernatant was extracted with two additional phenol-chloroform extractions and precipitated overnight at −20°C in isopropanol. The RNA was pelleted by centrifugation, washed with 70% ethanol, air-dried for 5 min at room temperature, and resuspended in nuclease-free water. Contaminating DNA was removed by DNase I (Ambion) digestion for 45 min at 37°C, followed by phenol-chloroform extractions, isopropanol precipitation, and resuspension of the total RNA in nuclease-free water, as described above. Bacterial mRNA was enriched using the MICROBExpress kit (catalog no. AM1905; Ambion), according to the manufacturer's recommendations. Duplicates of 5 μg of total RNA were used as an input for each sample, which were pooled in 30 μl nuclease-free water after precipitation. Depletion efficiencies were assessed using a Bioanalyzer platform (Agilent). In all cases, the 23S and 16S peaks had been reduced from 33 to 45% and 21 to 29% to 2.4 to 6.8% and 0.1 to 1.2%, respectively, of the total area to surface.

### Illumina transcriptome sequencing.

cDNA libraries were prepared from the depleted mRNA samples using the TruSeq RNA sample preparation kit (Illumina), according to the manufacturer's recommendations (low-throughput protocol starting with RNA fragmentation). The cDNA samples were each loaded for hybridization on one lane of an Illumina flow cell. Seventy-six base-pair sequencing was performed in paired-end mode with Illumina version 4 SBS reagents using the SCS version 2.6 data collection software. The raw image data were analyzed using the Illumina GA2 Pipeline version 1.6, with phasing corrections made against the standard PhiX control lane and all other parameters at their default. The reads were trimmed to 70 bp and the adaptor sequences filtered, as well as any reads with <90% of bases of score <Q20. These filtered reads were then aligned to the genome of B. thailandensis strain E264 (GenBank accession no. NC_007651 and NC_007650) using the TopHat software package. The significant analysis of microarrays (SAM) file was then run through an HTSeq-count script to extract the gene-level counts for the genes, as defined by the NCBI GenBank/gff file (GenBank-to-gff conversion was done with BioPerl). Finally, the read-level counts were compared with DESeq in R, with the in-built statistical analysis method based on negative binomial distribution (28). The average numbers of reads of the exponential- and stationary growth-phase samples were 33,162,087 and 34,752,164, respectively, whereas the average number of reads of the ceftazidime survivor samples was 34,336,756. The reads mapped to the 23S, 16S, and 5S genes were excluded from analysis.

### Parameter sweeps of mathematical models.

**FIG 1:**
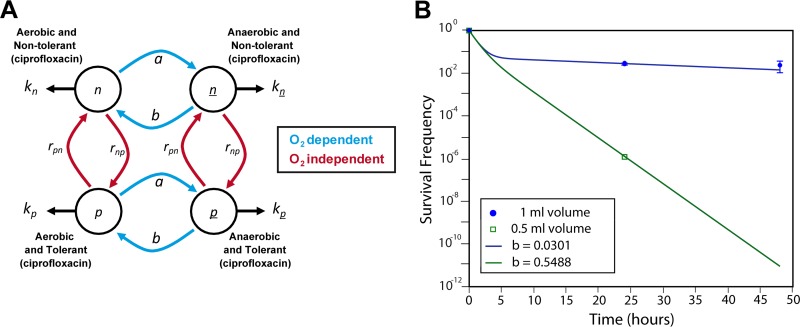
Four-state switching model. (A) The extended four-state model with (i) normal aerobic cells that are susceptible to ciprofloxacin and ceftazidime killing (*n*), (ii) aerobic cells that are tolerant to ciprofloxacin only (*p*), (iii) anaerobic cells that are susceptible to ciprofloxacin killing but tolerant to ceftazidime (*n*), and (iv) anaerobic cells that are multidrug tolerant (*p*). The different phenotypic population states are represented by circles and the switching rates between them by arrows. Under antibiotic treatment, a population state, *i*, declines (or grows if the drug is ineffective) at a rate *k_i_*. The transition rates between *n* and *p* and between *n* and *p* are hypothesized to be independent of the oxygen level and also the same (and so both sets are represented by *r_np_* and *r_pn_*). However, the switching rates *a* and *b* between *n* and *n* or *p* and *p* are oxygen dependent. (B) Four-state model fits ceftazidime killing data for the high and low survivor counts observed using different assay volumes *in vitro* (B). The four-state model is shown, with *a* = 0.0561 and *b* = 0.0301 (1-ml volume, blue circles) and *b* = 0.05488 (0.5-ml volume, green squares). The killing rates are the same in both runs with *k_n_* = *k_p_* = −1 and *k*_*n*_ = *k*_*p*_ = 0. The switching rates are *r_np_* = 0.02 and *r_pn_* = 0.1. These runs assume that the entire initial population was in the *n* state.

Solutions of the two-state stochastic switching model (see Fig. S4A in the supplemental material) were found using the analytical solution provided by Balaban et al. (9). The details of the results of the parameter searches are covered in the supplemental material. The numerical simulations of the three-state and four-state ordinary differential equation (ODE) models presented in Fig. 1B and also in Fig. S5 and S6 in the supplemental material were run in XPPAUT through a MatLab interface (MatLab R2011b; MathWorks, Inc., Natick, MA, USA). The parameter space search algorithms used here start in the regions of the parameter space and then gradually perturb the parameters as per a random walk by up to 1,000 steps. Each parameter set was used to calculate the resulting fit to the various objective data sets.

## RESULTS

### Large percentage of the stationary-phase population of B. pseudomallei and B. thailandensis survives exposure to high doses of ceftazidime under clinically relevant conditions.

**FIG 2:**
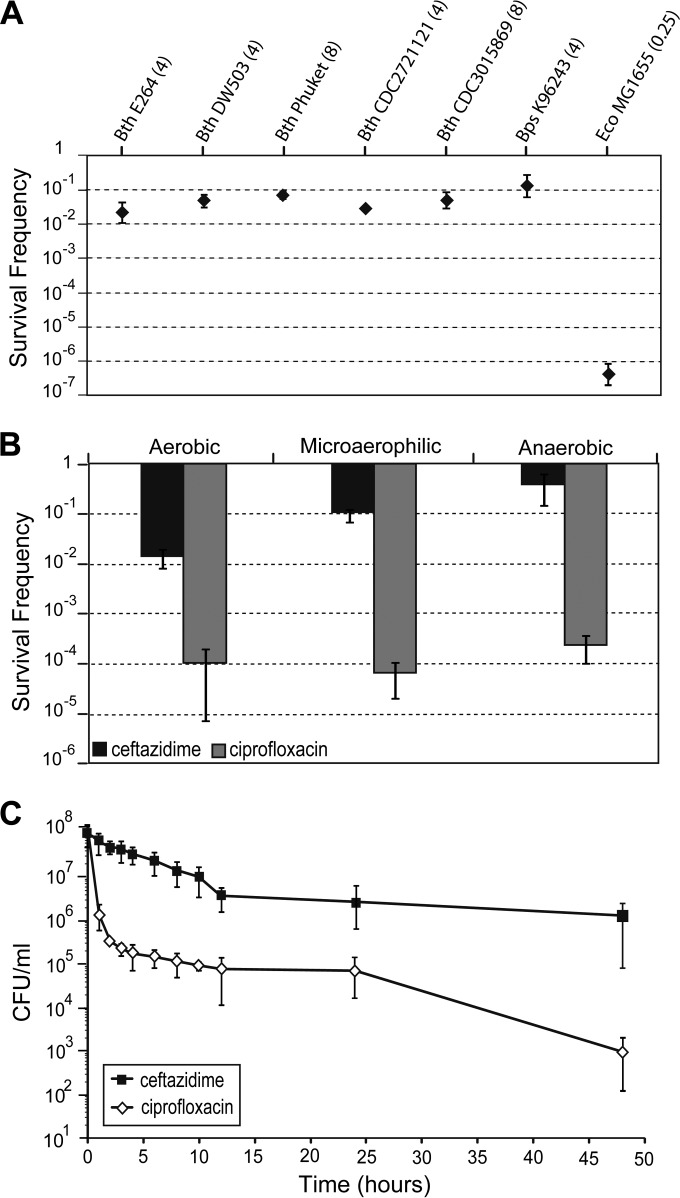
Persister levels and dynamics in Burkholderiales. (A) Survivor frequencies with various bacterial isolates. Aliquots of bacterial cultures were incubated in the presence of 100× the MIC of ceftazidime, and the surviving proportion was enumerated after 24 h incubation with antibiotics. Bth, B. thailandensis; Bps, B. pseudomallei; Eco, E. coli. The MIC of ceftazidime under atmospheric oxygen levels against each isolate is given in brackets after the strain designation. (B) Relationship between killing efficiencies of different antibiotics and oxygen levels. Aliquots of B. thailandensis strain E264 exposed to 100× the MIC of ceftazidime or 10× the MIC of ciprofloxacin were incubated in 24-well plates in different incubators: aerobic (atmospheric levels of oxygen), microaerophilic (5% O_2_), and anaerobic (no oxygen). The survival frequencies were assessed after 24 h incubation and are defined as the number of cells that survived the antibiotic treatment (*T*_24_) divided by the input CFU (*T*_0_). (C) Time-kill curves with two different antibiotics. Aliquots of bacterial cultures of B. thailandensis strain E264 were incubated in the presence of 100× the MIC of ceftazidime or 10× the MIC of ciprofloxacin under aerobic conditions. The surviving proportion was enumerated at the indicated time points and is plotted on a logarithmic scale over time. The error bars represent the standard deviation (SD) over the mean from at least three independent experiments.

Inside the host, bacteria able to establish chronic disease are proposed to survive in an equilibrium of replication and death, similar to the stationary phase of *in vitro* cultures (29). With this in mind, we have examined the ability of stationary-phase bacteria to resist killing by the β-lactam antibiotic ceftazidime, which is commonly used to treat melioidosis. Using a standardized persister cell assay that was designed to mimic slow-growing oxygen-limited *in vivo* conditions, we challenged cells from the stationary growth phase with antibiotics under static incubation (see Materials and Methods). We determined the survival rates of a panel of Burkholderia strains, including the pathogenic B. pseudomallei and nonpathogenic B. thailandensis isolates. The MICs of ceftazidime, measured in a microtiter tray assay under unshaken conditions, were similar for the B. thailandensis and B. pseudomallei isolates tested. E. coli strain MG1655 was included as a control organism in the assay. We observed similar survivor frequencies of between 10^−2^ and 10^−1^ with B. pseudomallei and B. thailandensis isolates in the presence of 100× the MIC of ceftazidime (Fig. 2A). In comparison, stationary-phase cultures of E. coli K-12 strain MG1655 returned survivors at a frequency of 10^−7^, indicating that Burkholderia survivors exhibit a higher tolerance to ceftazidime. We chose B. thailandensis strain E264, which is often used as a surrogate model for B. pseudomallei (30), for use in more detailed studies on persister cell formation.

### Tolerance to ceftazidime is oxygen dependent.

Previous work has shown that the oxygen levels in culture medium had a profound effect on the susceptibility of B. pseudomallei to antibiotics (31) We measured the killing of stationary-phase B. thailandensis by antibiotics under different oxygen availabilities. We observed an inverse relationship between ceftazidime killing and atmospheric oxygen levels (Fig. 2B). Killing by ciprofloxacin, a fluoroquinolone antibiotic that has been used less frequently to treat melioidosis (32), was not affected by atmospheric oxygen levels (Fig. 2B). We obtained similar results by changing the culture volume, which then changed the dissolved oxygen levels (see text and Fig. S5 in the supplemental material). Our results are in agreement with the findings by Hamad et al. (31), who demonstrated that some antibiotics, including ceftazidime, are less effective against B. pseudomallei cultured under anaerobic conditions.

In order to determine whether the ceftazidime survivors are a result of decreased drug efficacy under decreased oxygen levels or whether they can tolerate high doses of antibiotics for prolonged periods (5, 7, 33), the killing dynamics of stationary-phase cells exposed to ceftazidime or ciprofloxacin were assessed. We observed biphasic kill curves with a plateau of surviving cells after exposure to supra-MIC doses of either antibiotic (Fig. 2C). The initial killing rate was significantly higher for ciprofloxacin (Δlog CFU/ml/h = 2.57) than for ceftazidime (Δlog CFU/ml/h = 0.12). For both drug exposures, none of the colonies obtained at the 24-h time points (*n*, >200) grew on LB agar plates supplemented with 50 μg/ml ceftazidime or 20 μg/ml ciprofloxacin, showing that the survivors were not antibiotic-resistant mutants.

The ciprofloxacin survivors differed from the ceftazidime survivors in their regrowth rate after removal of the antibiotic. The ceftazidime survivors formed colonies of uniform size on LB agar plates after 24 h incubation at 37°C, whereas ciprofloxacin survivors required 48 h incubation before visible colonies of variable sizes were formed on the plates (see Fig. S1 in the supplemental material).

### Transcriptome profiling reveals distinct gene expression profile of ceftazidime survivors that is similar to that of oxygen-limited cells.

**FIG 3:**
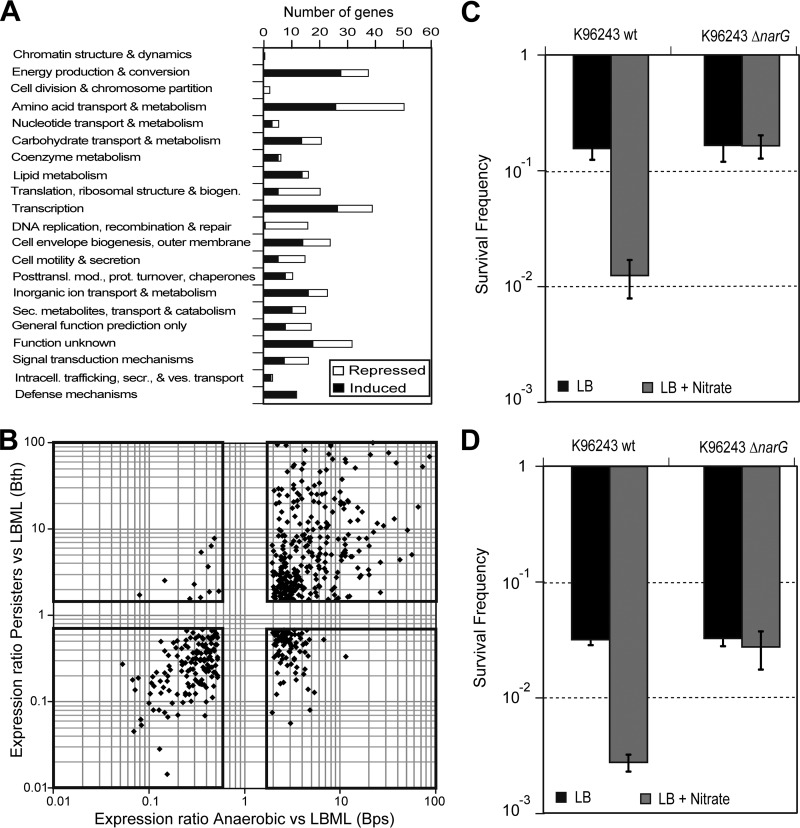
Functional classification of genes that are specifically induced and repressed in ceftazidime survivors and overlap hypoxia microarray data. (A) Functional annotation of genes that were induced in ceftazidime survivors compared to those in mid-log- and/or stationary-phase cells (black bars), and genes that were repressed in ceftazidime survivors compared to in mid-log- and/or stationary-phase cells (white bars). The functional categories were assigned according to the Burkholderia Genome Database (66). biogen., biogenetics; posttransl., posttranslation; mod., modification; prot., protein; sec./secr., secretory; ves, vesicle. (B) Correlation plot of expression ratios of B. pseudomallei (Bps) gene expression levels after 4 h anaerobic growth compared to those in LB mid-log cultures (microarray data by Hamad et al. [31]) and B. thailandensis (Bth) gene expression levels in ceftazidime survivors compared to those in LB mid-log cultures (RNA-seq data; this study). Each data point represents a gene that is found in both B. pseudomallei and B. thailandensis (*n* = 550). Genes that were commonly upregulated in both anaerobic B. pseudomallei samples and ceftazidime-tolerant B. thailandensis samples are displayed in the top right box, whereas genes that were commonly downregulated in both anaerobic B. pseudomallei samples and ceftazidime-tolerant B. thailandensis samples are displayed in the bottom left box. Only genes with an expression of ≥2 in the hypoxia data set and ≥1.5 in the persister data set are displayed. (C and D) Ceftazidime survivor frequencies of stationary-phase (C) or mid-log-phase (D) B. pseudomallei cultures incubated in the presence of 400 μg/ml ceftazidime in LB medium (black bars) or LB medium supplemented with or without 20 mM sodium nitrate (gray bars). Four independent biological replicates were used each with three technical replicates. The error bars indicate 1 SD. Asterisks denote significant differences between wild-type (wt) B. pseudomallei (K96243) and the Δ*narG* deletion mutant (*P* < 0.01).

To understand the physiological state of the drug-tolerant cells, we isolated stationary-phase B. thailandensis cells that had been grown unshaken in a normal (aerobic) atmosphere and then survived dosing with 100× the MIC of ceftazidime, and we sequenced their transcriptomes (see Materials and Methods). Comparing these to the transcriptome of mid-log-phase cells (LBML), a total of 1,292 genes showed >2-fold higher expression levels in the ceftazidime survivors, whereas the expression level of 1,171 genes was reduced by >2-fold in the ceftazidime survivors. Compared to stationary-phase cells (LBS), 1,372 genes were upregulated in the ceftazidime survivors, and 1,581 genes were downregulated ≥2-fold. Despite a similar number of differentially regulated genes, the coefficients of determination (*R*^2^) between the averages of ceftazidime survivors and those of the LBML samples and LBS samples were 0.751 and 0.004, respectively (see Fig. S1E and F in the supplemental material). This shows that the gene expression profile of ceftazidime survivors from stationary-phase cultures is more similar to the profile of actively growing cells. The coefficient of determination (*R*^2^) between the LBS and LBML samples was 0.002 (see Fig. S1D in the supplemental material). Cluster analysis indicated a low degree of commonality among the differentially regulated genes, with a high proportion of genes specifically expressed under one or two conditions (see Fig. S1G in the supplemental material). Our transcriptome analysis revealed a reduced level of expression of cell division and DNA replication genes in persisters, whereas defense mechanisms appeared to be upregulated (Fig. 3A). A comparison of the persister transcriptome to a recently published transcriptome of B. pseudomallei under hypoxia (31) revealed extensive similarities: 58% of the B. pseudomallei genes with an ortholog in B. thailandensis that were differentially expressed in cells under hypoxic conditions compared to those in aerobic cultures were differentially regulated in the same way in B. thailandensis persisters (Fig. 3B). This included the induction of the denitrification and arginine deimination (ADI) pathway (see text and Fig. S1H in the supplemental material), as well as the induction of F-type ATP synthase clusters on chromosome II, and a repression of a second ATP synthase cluster on chromosome I. Thirteen percent of the genes were inversely regulated in the two data sets. These included most flagellar and chemotaxis genes, which were induced in the stirred hypoxia samples and repressed in the static ceftazidime-challenged samples. This suggests that the assay conditions used here simulate a hypoxic environment and that the anaerobic metabolism of the ceftazidime survivors might relate to their antibiotic tolerance.

### B. pseudomallei narG plays a role in regulating persister cell frequency.

B. thailandensis and B. pseudomallei are obligate respirers and cannot grow under anaerobic conditions in the absence of an alternate electron acceptor, such as nitrate (31, 34). The treatment of B. pseudomallei with either ciprofloxacin or ceftazidime results in a biphasic kill curve similar to that seen in B. thailandensis. To test whether anaerobic nitrate respiration (denitrification) plays a direct role in persister cell formation, we constructed a B. pseudomallei narG deletion mutant (Δ*narG*) (see text and Fig. S9 in the supplemental material). The deletion of *narG* prevented anaerobic growth and caused a significant reduction in nitrate reductase activity under aerobic conditions (see Fig. S9B and D in the supplemental material). No aerobic growth defect was seen for the Δ*narG* mutant, which exhibited comparable aerobic growth rates to that of the wild type in both M9 minimal medium and L broth (see Fig. S9C and E in the supplemental material). Stationary-phase B. pseudomallei wild-type and Δ*narG* strains were dosed with ceftazidime, aerobically, in either LB medium or LB medium supplemented with 20 mM sodium nitrate. When stationary-phase wild-type or Δ*narG* bacteria were incubated with antibiotic in LB medium, the numbers of ceftazidime survivors were similar. However, in LB medium supplemented with nitrate, the Δ*narG* mutant formed drug-tolerant/persister cells at a higher frequency than did wild-type B. pseudomallei (Fig. 3C). The survivor frequency for mid-log-phase B. pseudomallei cultures was also assessed, showing nitrate to have a similar effect on wild-type but not mutant cultures (Fig. 3D). This points toward a role for anaerobic nitrate respiration and NarGHI in drug tolerance and susceptibility to ceftazidime.

### Modeling persister cell dynamics using a four-state model supports the existence of an anaerobic switch.

It has been proposed that stochastic switching between a faster growing phenotype, which is nontolerant to antibiotics, and a slower growing but drug-tolerant phenotype can account for the bacterial persistence phenomenon (9). This two-state switching model is depicted in Fig. S6A in the supplemental material. While this most parsimonious (i.e., simplest) model fitted some of the data from the original experiments, it did not explain the differences in survivors after exposure to ceftazidime or ciprofloxacin or the dependency on oxygen availability (Fig. 2B; see also Fig. S5B in the supplemental material). This suggests that there are at least three distinct phenotypes with different tolerances to these antibiotics. However, we found that neither a general three-state stochastic switching model (see Fig. S6B in the supplemental material) nor a modified three-state model that incorporates an oxygen-dependent switch (see Fig. S6C in the supplemental material) captured the kinetics of persister cell formation for both antibiotics, as described in the supplemental material.

We propose a solution by extending the switching model to four states composed of two separate stochastic switching mechanisms (Fig. 1A). The transition rates *a* and *b* control the switching of cells between aerobic and anaerobic metabolism, thereby controlling their tolerance to ceftazidime. These rates are assumed to depend on the oxygen content of the environment, whereas the rates *r_pn_* and *r_np_* are assumed to be independent of oxygen levels and control the tolerance of the cells to ciprofloxacin. This four-state model readily reproduces the invariable proportion of ciprofloxacin survivors while allowing oxygen levels to affect the survivor frequency under ceftazidime exposure over many orders of magnitude, as indicated by the fit to data in Fig. 1B. We present this four-state model as the minimal stochastic switching model necessary to reproduce all the features of the persistence phenomenon under different oxygen levels.

### Anaerobically adapted drug-tolerant cells can be eliminated by ciprofloxacin treatment or by introducing oxygen.

**FIG 4:**
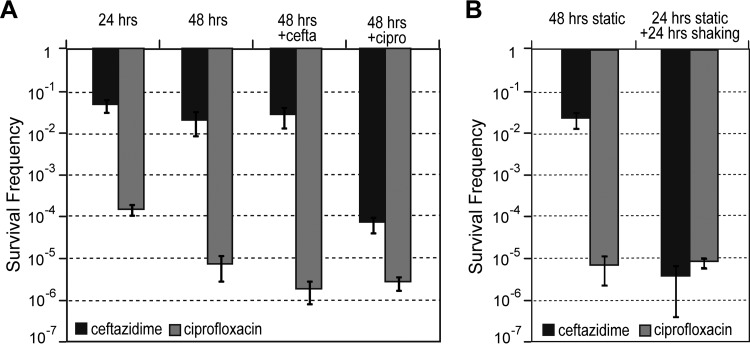
Elimination of anaerobic survivors. (A) Validation of the predicted antibiotic susceptibilities of different anaerobic persister populations was assessed by adding a second antibiotic to wells with bacteria that were previously challenged for 24 h with ceftazidime (cefta) (black bars) and ciprofloxacin (cipro) (gray bars). The survival frequencies were assessed after a further 24 h of incubation with the second antibiotic. Controls that underwent 48 h incubation with the initial antibiotic only were included for comparison. (B) Reversal of oxygen-dependent drug tolerance by introduction of oxygen was assessed by incubating 24-well plates containing bacteria that were previously challenged for 24 h with ceftazidime (black bars) and ciprofloxacin (gray bars), with aeration at 150 rpm. The survival frequencies were assessed after a further 24 h of incubation with shaking. Controls that underwent 48 h of static incubation were included for comparison. (A and B) Error bars represent the standard deviation over the mean values from at least three independent experiments.

The model described above predicts that the majority of the anaerobic cells are tolerant to ceftazidime but susceptible to ciprofloxacin (Fig. 1A, population *n*). To test this, the ceftazidime survivors were sequentially exposed to different combinations of ciprofloxacin or ceftazidime. The ceftazidime survivors were unaffected by the addition of more ceftazidime but were susceptible to killing by ciprofloxacin, with 273 times fewer survivors (*P* < 0.0001) than in the cells that had been treated for 48 h with one dose of ceftazidime alone (Fig. 4A). In contrast, the ciprofloxacin survivors were only marginally susceptible to further killing by ciprofloxacin (3 times fewer survivors; *P* = 0.014) and ceftazidime (4 times fewer survivors; *P* = 0.021), respectively, compared to cells that had been treated for 48 h with one dose of ciprofloxacin alone. The addition of the anaerobically active antibiotic metronidazole to the ceftazidime and ciprofloxacin survivors did not result in additional killing (data not shown).

Our model and our data on the formation of drug-tolerant cells by the Δ*narG* mutant predict that introducing oxygen or nitrate into the system would activate the aerobic metabolism in ceftazidime survivors and thus render them susceptible to killing by ceftazidime. To test this hypothesis, 24-well plates containing B. thailandensis cells previously challenged statically for 24 h with ceftazidime or ciprofloxacin were incubated for a further 24 h on a rotary shaker at 150 rpm. This resulted in a significantly lower survival frequency (*P* < 0.00001) of 3.8 × 10^−6^ in the case of ceftazidime-treated samples compared to 2.4 × 10^−2^ after static incubation for 48 h (Fig. 4B). No difference between the static and static plus shaking incubation conditions was observed with ciprofloxacin. These results indicate that the antibiotic tolerance due to anaerobic adaptation is reversible, and this type of drug-tolerant cells can be eradicated by introducing oxygen into the environment or by using antibiotics that can kill cells at low oxygen levels.

## DISCUSSION

Currently, there is much interest in noninherited antibiotic resistance and its role in bacterial disease and environmental survival. A characteristic feature of this type of resistance is a biphasic antibiotic killing curve, in which a fast initial kill phase is followed by much slower killing. The surviving cells are tolerant, rather than resistant (35), to antibiotics. Tolerance may arise as a result of reduced metabolic activity, lack of growth, and even dormancy of cells (3, 35, 36), and the term persister cells has been applied to dormant drug-resistant cells exhibiting a reduction in protein and ATP synthesis (3, 37–39). However, it is also clear that drug tolerance can arise as a consequence of mechanisms other than dormancy (40–42). Consequently, there are increasing numbers of reports of different types of drug-tolerant cells with different levels of metabolic activity and different patterns of drug tolerance (36, 40, 42–45).

The drug-tolerant cells we characterized retained metabolic activity, evidenced by our ability to map the transcriptome. This revealed the upregulation of a range of β-lactamases and efflux pumps in the drug-tolerant cells we isolated (see the data in the supplemental material). In addition, we found that several genes associated with the heat shock response were upregulated in the drug-tolerant cells. Of these, *htpX* and Hsp20 have been reported as being expressed in E. coli persister cells (39, 46). Overall, our findings suggest that the cells we isolated were drug-tolerant cells as a consequence of a change in their metabolism, rather than being dormant persister cells.

Against this background, for many decades, it has been known that compared to log-phase cells, stationary-phase cells are more resistant to β-lactam antibiotics (47). This drug tolerance is considered to be a consequence of the ability of β-lactams to target growing cells only. Consistent with this, persister cell frequencies increase as bacterial cultures enter the stationary growth phase (3, 33). Indeed, there are reports that with some species and strains, the entire stationary-phase culture becomes tolerant to β-lactam antibiotics (33).

In our studies, we have found that Burkholderia exposed to antibiotics under conditions similar to those likely to be experienced *in vivo* form drug-tolerant cells at frequencies of >2%, whereas under similar conditions, we found that E. coli formed drug-tolerant cells at a frequency of 10^−7^. For our studies, we have used bacteria from the stationary growth phase rather than the exponential growth phase because there is evidence that bacteria growing *in vivo* more closely resemble stationary-phase bacteria. A study on Haemophilus influenzae demonstrated that cells grown in animals adopt a physiological state that resembles late stationary-phase cultures *in vitro* (48). Likewise, the gene expression pattern of M. tuberculosis in starved stationary-phase cultures displayed features that characterize persistence *in vivo*, such as entry into a nonreplicative state and the metabolism of fatty acids (17). Transcriptional data previously obtained in our lab (49) also indicated that global gene expression in B. pseudomallei recovered from the lungs of infected mice is very similar to that of cells grown to stationary phase *in vitro*.

Another important finding from our work is that oxygen levels play a role in regulating the frequencies of drug-tolerant cells. An increasing number of studies show that oxygen-depleted environments constitute a common environment for bacterial pathogens that are able to establish chronic disease and also for environmental bacteria (50–54). It has been reported that the growth of Burkholderia bacteria under anaerobic conditions had a marked influence on the MICs of some antibiotics (31). In this study, we show that atmospheric oxygen levels influence the frequencies of cells tolerant to ceftazidime (but not ciprofloxacin). Our results also indicate that oxygen did not regulate E. coli persister cell frequencies, and this might reflect a difference in metabolic capacity. It will now be important to assess how oxygen levels influence persister cell frequency in other bacterial species.

The transcriptomes of bacteria that survived dosing with ceftazidime in unshaken broths and in a normal atmosphere revealed that their metabolism is more similar to cells grown under anaerobic conditions than to that of stationary-phase cells. This likely reflects a combination of the settling out of cells and the formation of an oxygen gradient in the medium. It is possible that the cells that settle out are in a relatively anaerobic environment, where they are unable to grow due to the lack of a terminal electron acceptor, such as oxygen or nitrate. It has been shown that even nongrowing bacteria require some form of cellular energy and redox balance to stay viable (55). Therefore, it is not surprising that survivors show upregulation of a range of genes encoding proteins required for anaerobic nitrate respiration (denitrification), including the NarGHI complex, which is required for the utilization of nitrate as a terminal electron acceptor.

The increased susceptibility of wild-type B. pseudomallei cells to ceftazidime with the addition of nitrate, which was not seen in the Δ*narG* mutant, implicates anaerobic nitrate respiration and NarGHI in drug tolerance. We propose that the increase in ceftazidime susceptibility with the addition of nitrate is due to the generation of a proton motive force (PMF) by NarGHI. Previous studies have implicated the generation of PMF by the addition of metabolites, such as nitrate, in antibiotic susceptibility (56, 57). Recently, a low abundance of NarGH in E. coli has been linked to aminoglycoside and cephalosporin (including ceftazidime) resistance, thought to be due to a lowered PMF (58). Increased aeration during the persister assay (Fig. 4B) resulted in an altered susceptibility of B. thailandensis cells to ceftazidime, potentially due to an increase in aerobic respiration. Both the addition of nitrate and the increased aeration during the persister assay are likely to initiate an active electron transport pathway, either via denitrification or oxidative phosphorylation, rendering Burkholderia bacteria susceptible to ceftazidime action. In summary, our work that has shown that drug tolerance is governed by anaerobic adaptation but not respiration.

Interestingly, the ceftazidime survivors obtained in our assay setup were not killed by the addition of metronidazole. This antibiotic has been shown to kill anaerobically adapted B. pseudomallei (31), and we observed the killing of 95% of the population of B. thailandensis within 24 h if incubated under strict anaerobic conditions in plain LB broth (data not shown). This suggests that either the anaerobic adaptation of the ceftazidime survivors was only partial or that the residual oxygen, or even the presence of ceftazidime, in the static incubation assay interfered with the activity of metronidazole. The effect of metronidazole in a model of chronic melioidosis remains to be elucidated.

We also used mathematical modeling to support our experimental results, and this modeling suggests that the different types of drug-tolerant cells we have seen result from the actions of two intertwined switches, one which involves oxygen-based metabolic switching. This modeling also predicted that the introduction of oxygen would eliminate persisters, and we confirmed this experimentally. Together with our results, these findings show that in many bacteria, including Burkholderia, drug tolerance is not necessarily due to the presence of a subpopulation of metabolically inactive cells (7, 40).

Our findings suggest new approaches to the control of melioidosis. Despite intensive antibiotic treatment, the mortality rates for melioidosis can be 40 to 50% in areas where the disease is endemic, and recurring disease occurs in 5 to 25% of cases (15, 59–61). Granuloma-like abscesses formed during chronic melioidosis might provide a hypoxic environment (15, 16, 62, 63), and bacteria that reside in hypoxic abscesses would not be targeted by ceftazidime treatment. Our results might suggest that ciprofloxacin or a combination therapy of ceftazidime and ciprofloxacin would be highly effective for the treatment of melioidosis, but previous studies have shown a variable efficacy for ciprofloxacin (32). This likely reflects the finding that some strains of B. pseudomallei show intermediate resistance to ciprofloxacin (64). Our finding that ceftazidime survivors lose their antibiotic tolerance after the addition of nitrate or the reintroduction of oxygen to the system might be another lead for improved success of chronic melioidosis treatment, through the elevation of respiratory activity. Hyperbaric oxygen treatment (HBO) is already successfully used in cases of chronic osteomyelitis and necrotizing infections (65). The effect of HBO or ozonized oxygen on melioidosis patients remains to be tested.

In summary, the findings of this study provide new insight into the mechanisms by which bacteria resist killing by antibiotics by modifying their metabolic state. It remains to be elucidated if these results and the oxygen-dependent switch in persister cell formation apply more generally to other bacterial species.

## Supplementary Material

Supplemental material

## References

[1] Bigger JW 1944 Treatment of staphylococcal infections with penicillin by intermittent sterilisation. Lancet 244:497–500. 10.1016/S0140-6736(00)74210-3

[2] Levin BR Rozen DE 2006 Non-inherited antibiotic resistance. Nat. Rev. Microbiol. 4:556–562. 10.1038/nrmicro1445 16778840

[3] Wood TK Knabel SJ Kwan BW 2013 Bacterial persister cell formation and dormancy. Appl. Environ. Microbiol. 79:7116–7121. 10.1128/AEM.02636-13 24038684 PMC3837759

[4] Mulcahy LR Burns JL Lory S Lewis K 2010 Emergence of Pseudomonas aeruginosa strains producing high levels of persiste cells in patients with cystic fibrosis. J. Bacteriol. 192:6191–6199. 10.1128/JB.01651-09 20935098 PMC2981199

[5] Fauvart M De Groote VN Michiels J 2011 Role of persister cells in chronic infections: clinical relevance and perspectives on anti-persister therapies. J. Med. Microbiol. 60:699–709. 10.1099/jmm.0.030932-0 21459912

[6] Lewis K 2007 Persister cells, dormancy and infectious disease. Nat. Rev. Microbiol. 5:48–56. 10.1038/nrmicro1557 17143318

[7] Lewis K 2010 Persister cells. Annu. Rev. Microbiol. 64:357–372. 10.1146/annurev.micro.112408.134306 20528688

[8] Jayaraman R 2008 Bacterial persistence: some new insights into an old phenomenon. J. Biosci. 33:795–805. 10.1007/s12038-008-0099-3 19179767

[9] Balaban NQ Merrin J Chait R Kowalik L Leibler S 2004 Bacterial persistence as a phenotypic switch. Science 305:1622–1625. 10.1126/science.1099390 15308767

[10] Lohmar I Meerson B 2011 Switching between phenotypes and population extinction. Phys. Rev. E. 84:051901. 10.1103/PhysRevE.84.051901 22181438

[11] Costerton JW Stewart PS Greenberg EP 1999 Bacterial biofilms: a common cause of persistent infections. Science 284:1318–1322. 10.1126/science.284.5418.1318 10334980

[12] Høiby N Ciofu O Johansen HK Song ZJ Moser C Jensen PØ Molin S Givskov M Tolker-Nielsen T Bjarnsholt T 2011 The clinical impact of bacterial biofilms. Int. J. Oral Sci. 3:55–65. 10.4248/IJOS11026 21485309 PMC3469878

[13] Saunders BM Britton WJ 2007 Life and death in the granuloma: immunopathology of tuberculosis. Immunol. Cell Biol. 85:103–111. 10.1038/sj.icb.7100027 17213830

[14] Conejero L Patel N de Reynal M Oberdorf S Prior J Felgner PL Titball RW Salguero FJ Bancroft GJ 2011 Low-dose exposure of C57BL/6 mice to Burkholderia pseudomallei mimics chronic human melioidosis. Am. J. Pathol. 179:270–280. 10.1016/j.ajpath.2011.03.031 21703409 PMC3123849

[15] Limmathurotsakul D Peacock SJ 2011 Melioidosis: a clinical overview. Br. Med. Bull. 99:125–139. 10.1093/bmb/ldr007 21558159

[16] Currie BJ Ward L Cheng AC 2010 The epidemiology and clinical spectrum of melioidosis: 540 cases from the 20 year Darwin prospective study. PLoS Negl. Trop. Dis. 4:e900. 10.1371/journal.pntd.0000900 21152057 PMC2994918

[17] Hampshire T Soneji S Bacon J James BW Hinds J Laing K Stabler RA Marsh PD Butcher PD 2004 Stationary phase gene expression of Mycobacterium tuberculosis following a progressive nutrient depletion: a model for persistent organisms? Tuberculosis 84:228–238. 10.1016/j.tube.2003.12.010 15207492 PMC3195342

[18] Folsom J Richards L Pitts B Roe F Ehrlich G Parker A Mazurie A Stewart P 2010 Physiology of Pseudomonas aeruginosa in biofilms as revealed by transcriptome analysis. BMC Microbiol. 10:294. 10.1186/1471-2180-10-294 21083928 PMC2998477

[19] Thomas AD Forbes-Faulkner JC 1981 Persistence of Pseudomonas pseudomallei in soil. Aust. Vet. J. 57:535–536. 10.1111/j.1751-0813.1981.tb05804.x 7342941

[20] Wuthiekanun V Smith MD White NJ 1995 Survival of Burkholderia pseudomallei in the absence of nutrients. Trans. R. Soc. Trop. Med. Hyg. 89:491–491. 10.1016/0035-9203(95)90080-2 8560519

[21] Pumpuang A Chantratita N Wikraipat C Saiprom N Day NPJ Peacock SJ Wuthiekanun V 2011 Survival of Burkholderia pseudomallei in distilled water for 16 years. Trans. R. Soc. Trop. Med. Hyg. 105:598–600. 10.1016/j.trstmh.2011.06.004 21764093 PMC3183224

[22] Robertson J Levy A Sagripanti JL Inglis TJ 2010 The survival of Burkholderia pseudomallei in liquid media. Am. J. Trop. Med. Hyg. 82:88–94. 10.4269/ajtmh.2010.09-0226 20065001 PMC2803515

[23] Yu Y Kim HS Chua HH Lin CH Sim SH Lin D Derr A Engels R DeShazer D Birren B Nierman WC Tan P 2006 Genomic patterns of pathogen evolution revealed by comparison of Burkholderia pseudomallei, the causative agent of melioidosis, to avirulent Burkholderia thailandensis. BMC Microbiol. 6:46. 10.1186/1471-2180-6-46 16725056 PMC1508146

[24] Godoy D Randle G Simpson AJ Aanensen DM Pitt TL Kinoshita R Spratt BG 2003 Multilocus sequence typing and evolutionary relationships among the causative agents of melioidosis and glanders, Burkholderia pseudomallei and Burkholderia mallei. J. Clin. Microbiol. 41:2068–2079. 10.1128/JCM.41.5.2068-2079.2003 12734250 PMC154742

[25] Logue CA Peak IR Beacham IR 2009 Facile construction of unmarked deletion mutants in Burkholderia pseudomallei using *sacB* counter-selection in sucrose-resistant and sucrose-sensitive isolates. J. Microbiol. Methods 76:320–323. 10.1016/j.mimet.2008.12.007 19150470

[26] Link AJ Phillips D Church GM 1997 Methods for generating precise deletions and insertions in the genome of wild-type Escherichia coli: application to open reading frame characterization. J. Bacteriol. 179:6228–6237 9335267 10.1128/jb.179.20.6228-6237.1997 PMC179534

[27] Chuang SE Daniels DL Blattner FR 1993 Global regulation of gene expression in Escherichia coli. J. Bacteriol. 175:2026–2036 8458845 10.1128/jb.175.7.2026-2036.1993 PMC204293

[28] Anders S Huber W 2010 Differential expression analysis for sequence count data. Genome Biol. 11:R106. 10.1186/gb-2010-11-10-r106 20979621 PMC3218662

[29] Nataro JP Blaser MJ Cunningham-Rundles S 2000 Persistent bacterial infections; commensalism gone awry or adaptive niche? p 3–12 *In* Nataro JP Blaser MJ Cunningham-Rundles S (ed), Persistent bacterial infections. ASM Press, Washington, DC

[30] Haraga A West TE Brittnacher MJ Skerrett SJ Miller SI 2008 Burkholderia thailandensis as a model system for the study of the virulence-associated type III secretion system of Burkholderia pseudomallei. Infect. Immun. 76:5402–5411. 10.1128/IAI.00626-08 18779342 PMC2573339

[31] Hamad MA Austin CR Stewart AL Higgins M Vázquez-Torres A Voskuil MI 2011 Adaptation and antibiotic tolerance of anaerobic Burkholderia pseudomallei. Antimicrob. Agents Chemother. 55:3313–3323. 10.1128/AAC.00953-10 21537012 PMC3122399

[32] Liew FY Tay ST Puthucheary SD 2011 Reduced susceptibility of Malaysian clinical isolates of Burkholderia pseudomallei to ciprofloxacin. Trop. Biomed. 28:646–650 22433895

[33] Keren I Kaldalu N Spoering A Wang Y Lewis K 2004 Persister cells and tolerance to antimicrobials. FEMS Microbiol. Lett. 230:13–18. 10.1016/S0378-1097(03)00856-5 14734160

[34] Andreae CA Titball RW Butler CS 2014 Influence of the molybdenum cofactor biosynthesis on anaerobic respiration, biofilm formation and motility in Burkholderia thailandensis. Res. Microbiol. 165:41–49. 10.1016/j.resmic.2013.10.009 24239959

[35] Lewis K 2007 Persister cells, dormancy and infectious disease. Nat. Rev. Microbiol. 5:48–56. 10.1038/nrmicro1557 17143318

[36] Balaban NQ Merrin J Chait R Kowalik L Leibler S 2004 Bacterial persistence as a phenotypic switch. Science 305:1622–1625. 10.1126/science.1099390 15308767

[37] Kwan BW Valenta JA Benedik MJ Wood TK 2013 Arrested protein synthesis increases persister-like cell formation. Antimicrob. Agents Chemother. 57:1468–1473. 10.1128/AAC.02135-12 23295927 PMC3591907

[38] Lewis K 2010 Persister cells. Annu. Rev. Microbiol. 64:357–372. 10.1146/annurev.micro.112408.134306 20528688

[39] Shah D Zhang Z Khodursky A Kaldalu N Kurg K Lewis K 2006 Persisters: a distinct physiological state of E. coli. BMC Microbiol. 6:53. 10.1186/1471-2180-6-53 16768798 PMC1557402

[40] Goneau LW Yeoh NS Macdonald KW Cadieux PA Burton JP Razvi H Reid G 2014 Selective target inactivation rather than global metabolic dormancy causes antibiotic tolerance in uropathogens. Antimicrob. Agents Chemother. 58:2089–2097. 10.1128/AAC.02552-13 24449771 PMC4023725

[41] Balaban NQ Gerdes K Lewis K McKinney JD 2013 A problem of persistence: still more questions than answers? Nat. Rev. Microbiol. 11:587–591. 10.1038/nrmicro3076 24020075

[42] Orman MA Brynildsen MP 2013 Dormancy is not necessary or sufficient for bacterial persistence. Antimicrob. Agents Chemother. 57:3230–3239. 10.1128/AAC.00243-13 23629720 PMC3697331

[43] Zhang Y Yew WW Barer MR 2012 Targeting persisters for tuberculosis control. Antimicrob. Agents Chemother. 56:2223–2230. 10.1128/AAC.06288-11 22391538 PMC3346619

[44] Allison KR Brynildsen MP Collins JJ 2011 Heterogeneous bacterial persisters and engineering approaches to eliminate them. Curr. Opin. Microbiol. 14:593–598. 10.1016/j.mib.2011.09.002 21937262 PMC3196368

[45] Luidalepp H Jõers A Kaldalu N Tenson T 2011 Age of inoculum strongly influences persister frequency and can mask effects of mutations implicated in altered persistence. J. Bacteriol. 193:3598–3605. 10.1128/JB.00085-11 21602347 PMC3133311

[46] Keren I Shah D Spoering A Kaldalu N Lewis K 2004 Specialized persister cells and the mechanism of multidrug tolerance in Escherichia coli. J. Bacteriol. 186:8172–8180. 10.1128/JB.186.24.8172-8180.2004 15576765 PMC532439

[47] Baich A 1969 Inhibitory effect of penicillin on proline synthesis in Escherichia coli. J. Bacteriol. 100:969–973 4901368 10.1128/jb.100.2.969-973.1969 PMC250182

[48] Dargis M Gourde P Beauchamp D Foiry B Jacques M Malouin F 1992 Modification in penicillin-binding proteins during *in vivo* development of genetic competence of Haemophilus influenzae is associated with a rapid change in the physiological state of cells. Infect. Immun. 60:4024–4031 1328054 10.1128/iai.60.10.4024-4031.1992 PMC257432

[49] Ooi WF Ong C Nandi T Kreisberg JF Chua HH Sun G Chen Y Mueller C Conejero L Eshaghi M Ang RM Liu J Sobral BW Korbsrisate S Gan YH Titball RW Bancroft GJ Valade E Tan P 2013 The condition-dependent transcriptional landscape of Burkholderia pseudomallei. PLoS Genet. 9:e1003795. 10.1371/journal.pgen.1003795 24068961 PMC3772027

[50] Gupta S Chatterji D 2005 Stress responses in mycobacteria. IUBMB Life 57:149–159. 10.1080/15216540500090611 16036577

[51] Rustad TR Sherrid AM Minch KJ Sherman DR 2009 Hypoxia: a window into Mycobacterium tuberculosis latency. Cell. Microbiol. 11:1151–1159. 10.1111/j.1462-5822.2009.01325.x 19388905

[52] Callaghan M McClean S 2012 Bacterial host interactions in cystic fibrosis. Curr. Opin. Microbiol. 15:71–77. 10.1016/j.mib.2011.11.001 22137884

[53] Williamson KS Richards LA Perez-Osorio AC Pitts B McInnerney K Stewart PS Franklin MJ 2012 Heterogeneity in Pseudomonas aeruginosa biofilms includes expression of ribosome hibernation factors in the antibiotic-tolerant subpopulation and hypoxia-induced stress response in the metabolically active population. J. Bacteriol. 194:2062–2073. 10.1128/JB.00022-12 22343293 PMC3318454

[54] de Beer D Stoodley P Roe F Lewandowski Z 1994 Effects of biofilm structures on oxygen distribution and mass transport. Biotechnol. Bioeng. 43:1131–1138. 10.1002/bit.260431118 18615526

[55] Boshoff HI Barry CE III 2005 Tuberculosis–metabolism and respiration in the absence of growth. Nat. Rev. Microbiol. 3:70–80. 10.1038/nrmicro1065 15608701

[56] Allison KR Brynildsen MP Collins JJ 2011 Metabolite-enabled eradication of bacterial persisters by aminoglycosides. Nature 473:216–220. 10.1038/nature10069 21562562 PMC3145328

[57] Borriello G Richards L Ehrlich GD Stewart PS 2006 Arginine or nitrate enhances antibiotic susceptibility of Pseudomonas aeruginosa in biofilms. Antimicrob. Agents Chemother. 50:382–384. 10.1128/AAC.50.1.382-384.2006 16377718 PMC1346784

[58] Ma Y Guo C Li H Peng XX 2013 Low abundance of respiratory nitrate reductase is essential for Escherichia coli in resistance to aminoglycoside and cephalosporin. J. Proteomics 87:78–88. 10.1016/j.jprot.2013.05.019 23711407

[59] Wiersinga WJ Scheepstra CG Kasanardjo JS de Vries PJ Zaaijer H Geerlings SE 2006 Dengue fever-induced hemolytic uremic syndrome. Clin. Infect. Dis. 43:800–801. 10.1086/507111 16912966

[60] Wiersinga WJ Currie BJ Peacock SJ 2012 Melioidosis. N. Engl. J. Med. 367:1035–1044. 10.1056/NEJMra1204699 22970946

[61] White NJ 2003 Melioidosis. Lancet 361:1715–1722. 10.1016/S0140-6736(03)13374-0 12767750

[62] Dance DA 2002 Melioidosis. Curr. Opin. Infect. Dis. 15:127–132. 10.1097/00001432-200204000-00005 11964912

[63] Puthucheary SD Sam IC 2012 Why is the response rate slow in ceftazidime therapy for melioidosis? Expert Rev. Anti Infect. Ther. 10:5–7. 10.1586/eri.11.158 22149608

[64] Sivalingam SP Sim SH Aw LT Ooi EE 2006 Antibiotic susceptibility of 50 clinical isolates of Burkholderia pseudomallei from Singapore. J. Antimicrob. Chemother. 58:1102–1103. 10.1093/jac/dkl359 16951412

[65] Cimsit M Uzun G Yildiz S 2009 Hyperbaric oxygen therapy as an anti-infective agent. Expert Rev. Anti Infect. Ther. 7:1015–1026. 10.1586/eri.09.76 19803709

[66] Winsor GL Khaira B Van Rossum T Lo R Whiteside MD Brinkman FS 2008 The Burkholderia Genome Database: facilitating flexible queries and comparative analyses. Bioinformatics 24:2803–2804. 10.1093/bioinformatics/btn524 18842600 PMC2639269

