# Supplementary material for: Bacterial Drug Tolerance under Clinical Conditions Is Governed by Anaerobic Adaptation but not Anaerobic Respiration
Source: Antimicrob Agents Chemother. 2014 Oct;58(10):5775–83. doi: 10.1128/AAC.02793-14 (PMC4187911; doi:10.1128/AAC.02793-14)
Supplement: Supplemental material [file supp_58_10_5775__index.html]

Bacterial Drug Tolerance under Clinical Conditions Is Governed by Anaerobic Adaptation but not Anaerobic Respiration — Supplemental material 

# Bacterial Drug Tolerance under Clinical Conditions Is Governed by Anaerobic Adaptation but not Anaerobic Respiration

## Supplemental material

**Files in this Data Supplement:**

- Supplemental file 1 -

  Additional experimental details, Supplemental Figures S1 to S10, and Tables S1 and S2.

  PDF, 1.7M
