# Supplementary material for: Bacterial Drug Tolerance under Clinical Conditions Is Governed by Anaerobic Adaptation but not Anaerobic Respiration
Source: Antimicrob Agents Chemother. 2014 Oct;58(10):5775–83. doi: 10.1128/AAC.02793-14 (PMC4187911; doi:10.1128/AAC.02793-14)
Supplement: Supplemental material [file AAC.02793-14_zac010143288so1.pdf]

**Hemsley *et al*. – Supplementary Information**

## Persister transcriptome.

To characterise the physiological state of drug-tolerant cells, the transcriptome of ceftazidime-survivors was sequenced. Samples were obtained by isolating RNA from stationary phase cells which had been treated with 100 x MIC ceftazidime for 24 hrs at 37 °C. The transcriptome was compared to the transcriptome of bacteria which had been grown in LB broth to mid-log phase ($OD_{600nm}$ = 0.5-0.6; abbreviated as LBML) or to stationary phase ($OD_{600nm}$ = 4.5-5.5; abbreviated as LBS). The reproducibility between biological replicates was assessed by linear regression analyses, where the coefficients of determination ($R^2$) ranged between 0.9905 and 0.9997 between biological replicates (Fig. S1A-C). The average number of reads per gene was 1,934 for mid-log phase samples (maximum of 292,702 reads). In contrast, the average number of reads per gene was 1,069 for ceftazidime-survivors, with a maximum of 191,687, which indicates that the overall gene expression level is reduced in ceftazidime-survivors compared to actively growing cells. In LBS samples, the average and maximum number of reads per gene were 1,927 and 1,093,163, respectively. The maximum number of reads dropped to 354,873, when BTH_I1628, encoding a small RNA with similarity to tmRNA, was excluded.

According to the transcriptome data, genes involved in nitrogen metabolism such as the nitric oxide reductase and the nitrate reductase (NarGHI) of the large chromosome, including the activators NarL and NarX (encoding a two component system), are induced in persister cells compared to both mid-log and stationary phase cells (Fig. S1H). *B. pseudomallei* (1) and *B. thailandensis* (2) are able to grow

anaerobically using nitrate as terminal electron acceptor. Induction of the nitrate reductases in persister cell samples is surprising as no nitrate was present during growth and challenge with antibiotics. Genes of the arginine deimination (ADI) pathway are the most highly up-regulated genes in persisters. The first enzyme of the ADI pathway, the arginine deiminase, converts L-arginine to L-citrulline and ammonia (Fig. S1H). Citrulline is degraded further forming ATP, $CO_2$, and L-ornithine (3). Hence, the ADI pathway provides energy in the absence of oxygen and also feeds into carbon and nitrogen metabolism. In addition, it has been suggested that the pathway can also protect some bacteria from acidic conditions, by the production of ammonia (4). Indeed, *Burkholderia pseudomallei* mutants in the ADI pathway have recently been described as exhibiting decreased survival rates at low pH (5). The same authors described increased expression of the *arcA* and *arcC* genes in certain colony morphotypes, but could not demonstrate a role for the ADI pathway in survival in macrophages. *B. thailandensis* does not form colony morphology variants to the same extend as *B. pseudomallei*. Nevertheless, the results substantiate a fitness advantage of cells with an active ADI pathway. The role of the arginine deimination pathway in virulence in animal models and chronic disease remains to be elucidated.

The promoter prediction tool Bprom (freely available at the Softberry homepage) predicted binding sites of the transcriptional regulator Fnr in vicinity of some promoters within the denitrification pathway and the ADI pathway operons (see Fig. S1H). Fnr has been extensively studied in *E. coli*, where it mediates the transition from aerobic to anaerobic growth (6). *B. thailandensis* strain E264 possesses three proteins with homology to the *E. coli* Fnr protein, BTH_II0035, BTH_II0460, and

BTH_II1244. The first two proteins were expressed at slightly higher levels in persisters compared to LBML samples only. However, Fnr activation in response to hypoxia is achieved through changes in the redox state of an internal iron-sulfur cluster (7), and not through transcriptional activation. The role of Fnr in the anaerobic adaptation in *Burkholderia* remains to be established.

The download link for the persister datasets is:
http://osslab.ex.ac.uk/downloads/RNAseq-data_Hemsley_et_al.xls

**Comparison with the transcriptomes of *M. tuberculosis* and *E. coli* persister cells.**

In order to identify commonalities between persisters cells from different organisms, the *B. thailandensis* transcriptome described here was compared to reported microarray data from *M. tuberculosis* persisters (6) and *E. coli* persisters (7, 8). This identified 16 genes that were commonly induced in drug-tolerant *B. thailandensis* and in *M. tuberculosis* persister cells (Fig S2). Seven of the genes encode predicted inner membrane proteins, and one conserved hypothetical protein of *M. tuberculosis*, Rv0140, has been found in culture filtrates (9). Two transcriptional regulators are among the list of commonly induced genes, and a variety of stress-related gene product such as heat-shock proteins and a universal stress protein. The latter, however, was only found to be up-regulated in persisters compared to mid-log phase cells but not to stationary-phase cells, thereby highlighting the effect of the reference sample in such transcriptome analyses. Only two of the commonly induced genes, the heat shock protein *htpX* and a member of the Hsp20 family, have also been described as being expressed in *E. coli* persisters, thereby

indicating a possible role of the heat shock response in persister cell formation across various organisms. Interestingly, over-expression of *hspX* in *M. tuberculosis* has been shown to result in reduced growth rates both *in vivo* and *in vitro* (10), thereby adding evidence to a possible role of HspX in maintaining a slow-growing phenotype.

**Role of the heat-shock response.**

We could not confirm activation of the heat-shock response in drug-tolerant cells (Fig S3). The heat shock sigma-factor *rpoH* was expressed at 3-times higher levels in drug-tolerant cells compared to both mid-log and stationary phase cells. The heat shock proteins HtpX (BTH_I0131) and two members of the Hsp20 family (BTH_I2809 and BTH_I2810), as well as the chaperone protein DnaK (BTH_I1308) were also induced in persisters. We also found elevated expression levels of some ATP-dependent proteases such as Lon and Clp proteases in drug-tolerant cells compared to mid-log phase cells, but not compared to stationary phase cells. Only one copy of the Zn-dependent oligopeptidase PrlC (BTH_I1860) was 2.4-fold induced in drug-tolerant cells. Finally, BTH_I2241 encoding the FtsH protease activity modulator HflK, and its downstream gene were up-regulated in drug-tolerant cells compared to stationary phase cells. FtsH has been shown to regulate RpoH levels and is important for maintaining membrane integrity (11). In *M. tuberculosis*, FtsH is involved in resistance to reactive oxygen species and regulates the cell division protein FtsZ (12) and in *C. crescentus*, *ftsH* mutants exhibit increased sensitivity to antibiotics and form filaments (13).

**Contribution of β-lactamases and multidrug efflux pumps.**

According to our RNAseq data, four putative β-lactamases, BTH__1908, BTH_II0372-0373, and BTH_II1450, were induced in drug-tolerant cells (Fig S4A). The latter represents a homologue of PenA, a secreted β-lactamase that increases resistance to β-lactam antibiotics when over-expressed in *B. pseudomallei* (14). However, we found that the remaining ceftazidime concentration after 24 hrs incubation at 37°C in the absence and presence of bacteria was approx. 95% of the initial concentration (i.e. 95x MIC) in both cases, thereby ruling out loss of activity by natural or bacterial degradation (see Fig S4 B&C). The expression of several multidrug-efflux pumps, including the BpeAB-OprB systems and the AmrAB-OprA system, was induced in persister (Fig S4A).

**Comparison of the Two, Three and Four State Stochastic Switching models.**

This section combines arguments, data and simulations to describe the relative effectiveness of the two, three and four state models in accounting for the data presented in the main text. In addition to changing the atmospheric oxygen levels, we also determined the number of persister cells in response to differences in the volume of the culture. Because these cultures were unshaken, and considering the diffusion of oxygen into water and broth was governed similarly, we were able to predict the oxygen levels in the broth in response to differences in culture volume (Fig S5A). Figures 1B and S5B demonstrate that the number of bacterial survivors under 24 hrs of ceftazidime treatment varied over about five orders of magnitude between conditions of high and low oxygen supply. However, in both experiments where oxygen levels were altered, either being explicitly controlled or when it was varied indirectly by changing the assay volume, the frequency of survivors revealed after 24 hrs of ciprofloxacin treatment stayed consistently at around $10^{-4}$.

***Two-State Model***. The two-state model (see Fig. S6A) proposes that there are two phenotypically distinct states, one with (*p*) and one without (*n*) antibiotic tolerance. Cells are assumed to stochastically switch between these states at rates *a* and *b*. As seen in Figure 1C, kill curves for both ciprofloxacin and ceftazidime exposure showed biphasic killing. These persister subpopulations however plateau at significantly different levels. Under ciprofloxacin treatment, the slope of the second phase (corresponds to the death rate $k_p$) was not very steep. This, combined with the consistency in survivor numbers to ciprofloxacin treatment after 24h under different oxygen regimes, strongly suggests that the switching rates (*a* and *b*) are not changing if *p* is to represent the ciprofloxacin-tolerant persister state. However, the very opposite is suggested under exposure to ceftazidime, where the killing efficacy changes many orders of magnitude with oxygen levels (Fig. S5B). The largest change that needs to be accounted for from the experiments is the difference observed by changing the assay volume from either 0.25 ml or 0.5 ml to 1 ml or 2 ml.

Using the analytical solution for the two-state switching model derived by Balaban *et al*. (15), we tested extensive regions of the parameter space summarised in Table S2 to match the killing data for ceftazidime and ciprofloxacin in various regimes. While some of these ranges appeared to overlap, a closer examination revealed that the parameter ranges must vary in order to account for the three separate killing regimes as illustrated in Figure S7 for the switching rates *a* and *b* when considered in tandem. In particular, comparing the ceftazidime treatment regimes revealed that the switching rates between the two states must change in order to account for the data. This is further illustrated in Figure S8, which plots two cases in which it is

assumed that $k_p = 0$ so that the other three parameters can be plotted. Very clear parameter changes are indicated by the separation of the parameter sets between high and low survivor numbers under ceftazidime treatment at different volumes. Thus the consistency of survivors from the ciprofloxacin treatment suggests there needs to be at least one additional subpopulation and hence a phenotypic state in the model. We conclude that at least a three-state stochastic switching model is required to explain the data.

***Three-State Model.*** The most general three-state stochastic switching model is presented in Figure S4B, with presumably two persister states $p_1$ and $p_2$ in addition to the fully susceptible $n$ state. For convenience, we chose the $p_2$ persisters to be those tolerant to ciprofloxacin. Kill curve data and the oxygen controlled assays (whether through volume or direct oxygen control) indicate that the killing of this subpopulation is seemingly independent of the oxygen content. This suggests that, independent of the oxygen concentration, this subpopulation should only be reduced to about $10^{-4}$ after 24 hrs of ciprofloxacin treatment. We know that the number of ceftazidime persisters is dependent on the oxygen level available. Thus the rates $a$ and $b$ are likely to vary under different oxygen conditions as the population size of anaerobic cells ($p_1$) needs to be mediated by oxygen. Therefore, we conclude that the switching rates in Figure S4B have the property that $f=d$ and $e=c$ (giving us the new model in Fig. S6C). Otherwise the consistency in the number of cells surviving ciprofloxacin treatment would also be oxygen dependent. To confirm this argument, parameter regimes of the ODE model presented in Figure S6C were extensively searched for solutions that could fulfil both the variability in ceftazidime-killing under different oxygen conditions and the consistent ciprofloxacin-killing. In each search,

the rates *c* and *d* were fixed to the best possible fit to the kill curve of ciprofloxacin at a 1 ml assay volume (see Fig. S5B). The other rates were then seeded over varying ranges and random walks were conducted of up to 1,000 steps searching for solutions which would fulfil the requisite high and low survivor numbers under ceftazidime-killing at various assay volumes. In no cases was a parameter set found which could even approach the total low number of survivors that a 0.5 ml assay volume would find (between $7.6 \times 10^{-7}$ and $1.7 \times 10^{-6}$ after 24 hrs ceftazidime). This is not surprising as the necessarily non-zero rates of *c* and *d* cause a buffering effect. These simulations did assume that the ciprofloxacin-tolerant persisters possess a similar tolerance to the ceftazidime drug as the anaerobic cells. The model could account for the data if it is assumed that the drug tolerance of the ciprofloxacin-tolerant cells with respect to ceftazidime exposure is oxygen dependent. However the model then loses any real explanatory or predictive power and becomes a purely fitting based exercise with no indication of how the behaviour may or may not change under different conditions.

Thus while the three-state model can capture some of the features of the experiments, it is unable to simultaneously capture all of the possible killing under different conditions while including all the features which have been indicated to be present from the experiments. The most evident being the independence of the number of ciprofloxacin survivors from oxygen conditions.

***Four State Model.*** The four-state model presented in Figure 3A proposes the incorporation of two stochastic switches into each cell. The switching rates between ciprofloxacin non-tolerance (*n* or *n*) and tolerance (*p* or *p*) is assumed to be

independent of oxygen. Thus, independent of the oxygen content of the environment, the number of ciprofloxacin persisters which survive treatment is consistent. However the other switch between a presumably aerobic or anaerobic metabolism is assumed to depend on the oxygen level of the environment. This new model can now fit all the data we have presented as indicated in Figure 3B. Indeed, in that example, only the switch out rate *b* from the anaerobic state needed to be modified to account for the different killing profiles observed under different levels of oxygen.

## ***Burkholderia pseudomallei*** **anaerobic nitrate respiration (denitrification)**

*B. thailandensis* and *B. pseudomallei* can grow anaerobically in the presence of nitrate (1, 2), using the denitrification pathway. Denitrification requires a series of reductase enzyme; nitrate reductase (NarGHI), nitrite reductase (Nir), nitric oxide reductase (Nor) and nitrous oxide (Nos), to sequentially reduce nitrate to dinitrogen gas (16). The first step in the denitrification (also referred to as anaerobic nitrate respiration pathway) utilises a membrane-bound nitrate reductase (NarGHI), required for the reduction of nitrate to nitrite and generation of a PMF (17). *B. pseudomallei* encodes two membrane-bound nitrate reductase enzymes *narGHJI* (BPSL2309-2312), predicted to be the main Nar required for anaerobic respiration and *narZYWV* (BPSS1156-1159) displaying homology to the cryptic NarZYWV found in *E. coli* and *Salmonella.* Both of these gene clusters share high sequence identity (90-99%) with respective homologous gene clusters in *B. thailandensis* BTH_I1851-BTH_I1854 (*narGHJI*) and BTH_II1249-1252 (*narZYWV*). The BTH_I11851-1854 gene cluster was shown to be upregulated in persister cells, whereas the second nitrate reductase BTH_II1249-1252 (*narZYWV)* was downregulated.

In order to determine the role of anaerobic respiration in persister cell formation a *narG* (BPSL2309) deletion mutant was constructed using the pDM4 suicide vector. The deletion of *narG* was confirmed using two sets of PCR, one using primers binding to a 300 bp internal region of the gene (not shown) and one using primers binding 300 bp up and downstream of the target gene, generating a 600 bp product in the mutant and a much larger product (approximately 3,500 bp) in the wild-type. No wild-type PCR product was detected under the cycle conditions used in this study (Fig. S9A). Deletion of *narG* (Δ*narG*) prevented growth of *B. pseudomallei* anaerobically in the presence of nitrate, but did not affect aerobic growth in either M9 minimal media or L-broth (Fig. S9 B – C and E). In order to confirm the Δ*narG* mutant was deficient in Nar activity a Griess reaction (Promega) was performed on aerobically grown wild-type and mutant *B. pseudomallei* cultures (grown in M9 minimal media supplemented with 20 mM sodium succinate and 20 mM sodium nitrate). The concentration of nitrite produced during aerobic growth and therefore relative Nar activity was determined using the Griess reaction and a nitrite standard curve. Only the wild-type accumulated significant amounts of nitrite after 8 hours of growth under aerobic conditions (Fig. S9D). After 24 hours of aerobic growth in the presence of nitrate wild-type *B. pseudomallei* accumulated around 256 µM nitrite whereas the Δ*narG* mutant accumulated only 7 µM nitrite. This indicated that NarGHI may be active under both aerobic and anaerobic conditions. The results from these experiments indicate BPSL2309-2312 (*narGHJI*) encodes the main nitrate reductase in *B. pseudomallei* required for anaerobic respiration and nitrate reductase activity.

**<u>Regrowth rates and phenotypes on plates</u>**

We observed differences in the re-growth rates between ceftazidime and ciprofloxacin-survivors (see Fig. S10), with the former forming colonies of uniform size on LB agar plates after 24 hrs incubation at 37 °C, whereas ciprofloxacin survivors requiring 48 hrs incubation before visible colonies of variable sizes are formed on plate. This suggests that ceftazidime-survivors seem to be able to rapidly resume growth, which might be the result of the maintained metabolic activity. The differences in the re-growth rates between ciprofloxacin- and ceftazidime survivors also indicate that they might have reached different dormancy levels. It has previously been suggested that dormancy levels can be divided into three stages, which coincide with arrests of growth, DNA replication, and protein synthesis, respectively, and that these levels correlate with resistance to different classes of antibiotics i.e. β-lactams, quinolones, and aminoglycosides (18).

## SUPPLEMENTARY METHODS

**Anaerobic growth experiments.** *B. pseudomallei* anaerobic growth studies were conducted using the BD GasPak EZ incubation system and two Gas Pak EZ anaerobic container system sachets with indicator. M9 minimal media supplemented with nitrate was used for the anaerobic growth studies and Griess reaction. M9 minimal media was supplemented with or without sodium nitrate ($NaNO_3^-$) or sodium nitrite ($NaNO_2^-$) (0-20 mM) and 20 mM succinate as a carbon and electron source. M9 media contained 2 mM $MgSO_4$, 0.1 mM $CaCl_2$, 20 % M9 salts (5 x stock solution; 85.5 gl-1 $Na_2HPO_4$, 15 gl-1 $KH_2PO_4$, 2.5 gl-1 NaCl, 5 gl-1 $NH_4Cl$). Wild-type *B. pseudomallei* and Δ*narG* overnight cultures were standardised and inoculated into M9 minimal media supplemented with or without 20 mM sodium nitrate. CFU counts

were performed by spot plating serial dilutions onto LB agar plates incubated aerobically at 37 $^{o}$C.

**Nitrate reductase activity assay.** The concentration of nitrite produced throughout aerobic growth in M9 minimal media was measured using the Griess Reagent system (Promega). 1 mL samples were taken throughout the growth cycle and frozen at -80 $^{o}$C prior to performing the Griess reaction. A nitrite standard curve was generated for each experiment to ensure accurate estimations of nitrite concentration in the sample medium. Three independent biological replicates were used, each with three technical replicates.

## SUPPLEMENTARY FIGURES

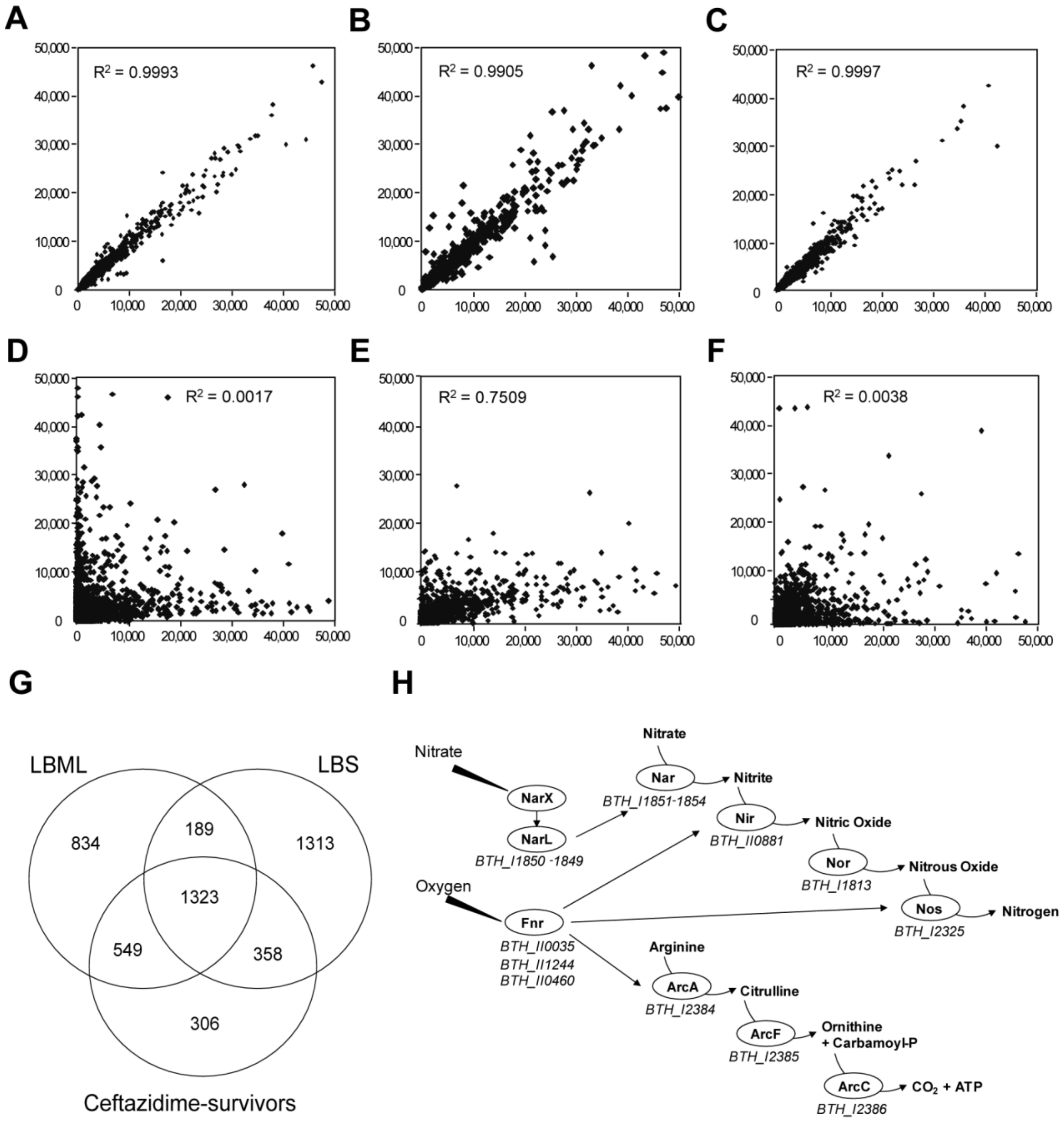


**Fig. S1. RNAseq data. (A-C)** Reproducibility between biological replicates. Representative scatter plots of read count data of two biological replicates of LBML samples (A), LBS samples (B), and ceftazidime-survivors (C), respectively. **(D-F)** Comparison of different conditions. Scatter plots of average read counts of LBS vs. LBML samples (D), ceftazidime-survivors vs. LBML samples (E), and ceftazidime-survivors vs. LBS samples (F), respectively. In A-F, coefficients of determination ($R^2$)

were calculated based on linear regression analyses. **(G)** Numbers within the Venn diagram represent genes that are specifically expressed under one or two conditions, or which are equally expressed under all three conditions (center of diagram). **(H)** Pathway analysis of the 306 genes that were specifically expressed in ceftazidime survivors revealed that all genes within the nitrate respiration (denitrification) pathway and the arginine deimination pathway were significantly upregulated in persisters compared to LBML and LBS samples. These pathways are controlled by various external stimuli through transcriptional regulators.

| Protein function | Bt-P/ML | Bt-P/S | TB-P | EC-P | Bt ID | TB ID | Ec ID | Localisation |
|---|---|---|---|---|---|---|---|---|
| Heat shock protein HtpX | | | | | BTH_I0131 | Rv0563 | *htpX* | IM |
| Sulphate permease family of anion transporters | | | | | BTH_I1051 | Rv1707 | *ychM* | IM |
| Peptidase, M50 family / conserved transmembrane protein | | | | | BTH_I1903 | Rv2625c | *rseP** | IM |
| Transcriptional regulator, MerR family | | | | | BTH_I2208 | Rv3334 | *zntR* | CP |
| Transcriptional regulator, ArsR family | | | | | BTH_II0561 | Rv2034 | *arsR* | CP |
| Alanine dehydrogenase / pyridine nucleotide transhydrogenase | | | | | BTH_II0922 | Rv2780 | *pntA* | IM |
| Heat shock protein, HSP20 family | | | | | BTH_II0924 | Rv0251c | *ibpB* | CP |
| Universal stress protein, putative | | | | | BTH_II1268 | Rv2623 | *uspE* | CP |
| AMP-binding domain protein / put. acyl-coA synthase / carnitine-CoA ligase | | | | | BTH_II1686 | Rv3515c | *caiC* | CP |
| Domain of unknown function / conserved hypothetical protein | | | | na | BTH_II1714 | Rv0140 | na | Secreted |
| Methylmalonate-semialdehyde dehydrogenase / phenylacetaldehyde dehydrogenase | | | | | BTH_II1801 | Rv0753c | *feaB* | CP |
| Pyochelin synthetase / conserved hypothetical protein | | | | | BTH_II1828 | Rv0100 | *entF* | CP/IM |
| 4-Aminobutyrate aminotransferase / put. lysine-(epsilon) aminotransferase | | | | | BTH_II2119 | Rv3290c | *puuE* | CP |
| Proline iminopeptidase / predicted aminoacrylate hydrolase | | | | | BTH_II2193 | Rv0840c | *rutD** | CP |
| 2-Oxoisovalerate dehydrogenase subunit / pyruvate dehydrogenase E1 | | | | na | BTH_II2304 | Rv2497c | na | CP |
| Sulphate permease family of anion transporters | | | | | BTH_II2316 | Rv1707 | *ychM* | IM |
| Stress response protein / HSP20 family | | | | na | BTH_II2321 | Rv0251c | na | IM |

* partial

**Fig. S2. Common themes in persister cell transcriptomes**. Transcriptome datasets of *B. thailandensis* persisters (Bt; this study), *M. tuberculosis* persisters (TB, Keren *et al.* 2011), and *E. coli* persisters (Ec; Keren *et al*. 2006) were analysed for similarities. Expression ratios refer to Bt persisters vs. mid-log cultures (Bt-P/ML) and stationary phase cultures (Bt-P/S), respectively, TB persisters revealed with D-cycloserine vs. mid-log phase cells before antibiotic challenge (TB-P), and *E. coli* persisters revealed with ampicillin vs. mid-log phase cells before antibiotic challenge (Ec-P). The genome of *B. thailandensis* was searched for orthologues of reported TB persister genes using BLASTP and the RNAseq dataset was analysed for expression ratios of these 17 homologues. Note that one TB gene, Rv1707, has two orthologues in *B. thailandensis*. Next, the genome of *E. coli* strain MG1655 was searched for orthologues of the common persister genes using BLASTP and a published microarray dataset (Keren *et a*l. 2006) was analysed for expression ratios in *E. coli*. A second *E. coli* dataset (Shah et al. 2006), which also includes a comparison of persisters vs. stationary phase cells, was also analysed; none of the orthologues exhibited induced expression levels. Details on the function and subcellular localisation of the encoded protein were obtained from public databases.

Colour coding for expression ratios: orange = ratio $< 0.5$; grey = ratio 0.5 to 2; green = ratio $>2$.

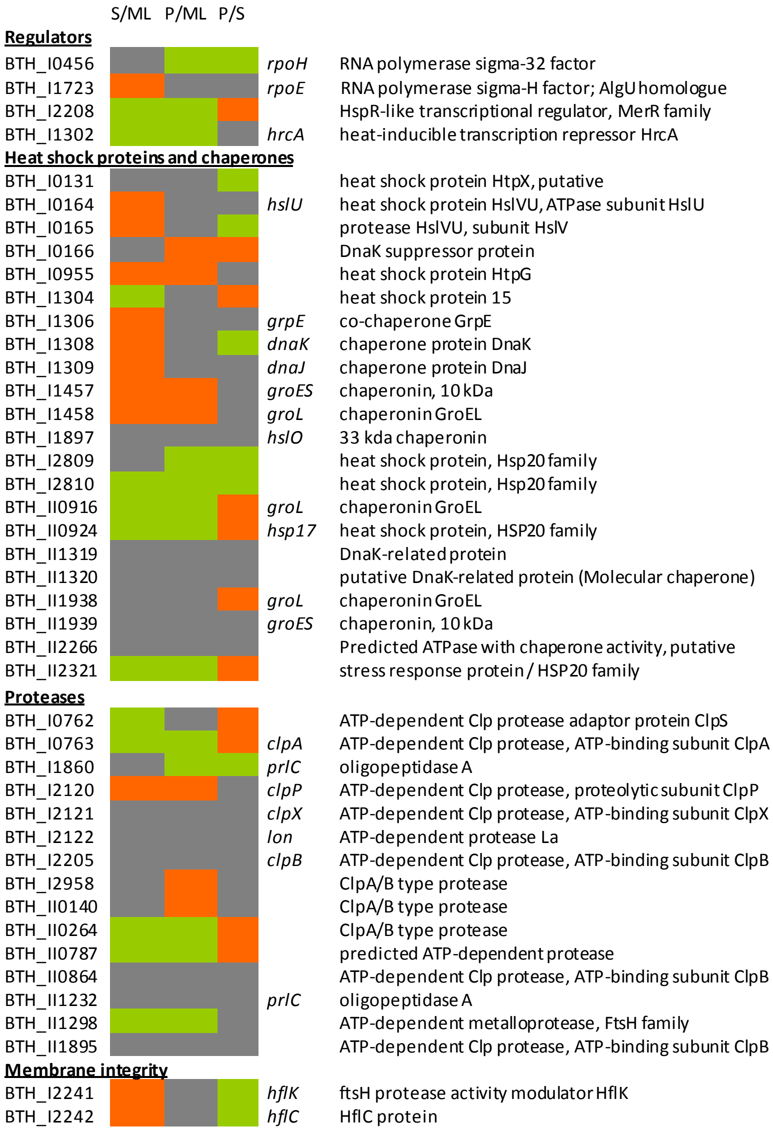


**Fig S3. Expression of the heat shock regulon in *B. thailandensis* persister cells**. Heat map of expression ratios of genes described to be part of the heat shock response in stationary phase samples vs. mid-log samples (S/ML), persisters vs. mid-log samples (P/ML) and persisters vs. stationary phase samples (P/S). Colour coding: orange = ratio < 0.5; grey = ratio 0.5 to 2; green = ratio >2.

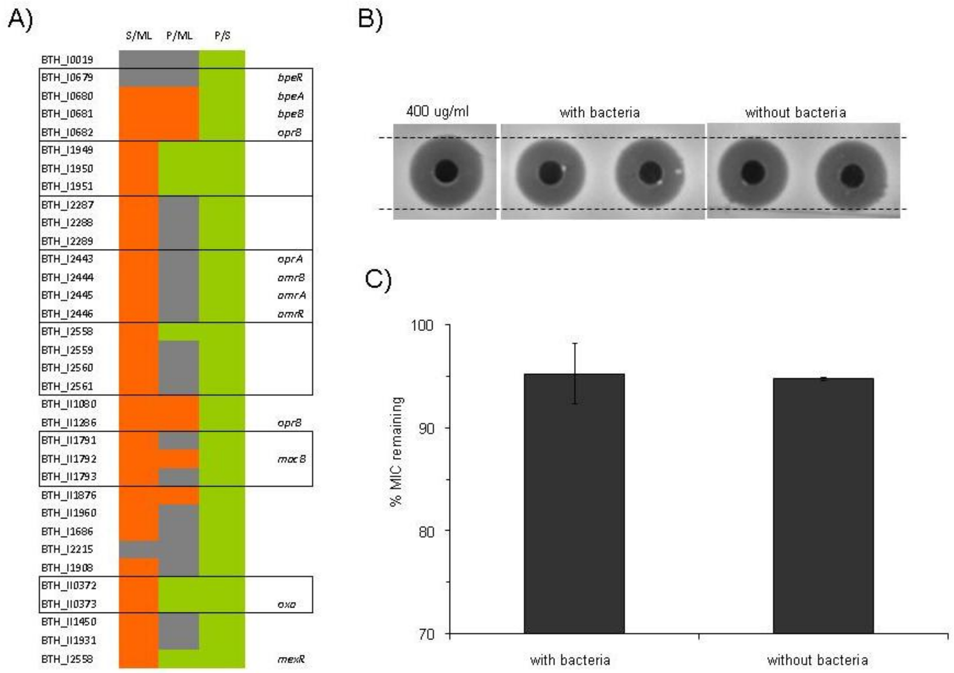


**Fig S4. Contribution of multidrug efflux systems and ß-lactamases**. A) Heat map of expression ratios of selected multidrug efflux systems in stationary phase samples vs. mid-log samples (S/ML), persisters vs. mid-log samples (P/ML) and persisters vs. stationary phase samples (P/S). Colour coding: orange = ratio < 0.5; grey = ratio 0.5 to 2; green = ratio >2. B-C) Remaining ceftazidime concentration after 24 hrs incubation with or without bacteria determined by a well diffusion assay. Zone of inhibition of ceftazidime standard at 400 µg/ml (same as used in persister assay), persister assay supernatants (with bacteria), or mock persister assay without bacteria (B) and results of measurements of the diameter of the zone of inhibition in the test samples relative to the 400 µg/ml standard (C). Bars and error bars in C) represent average and standard deviations of three independent samples.

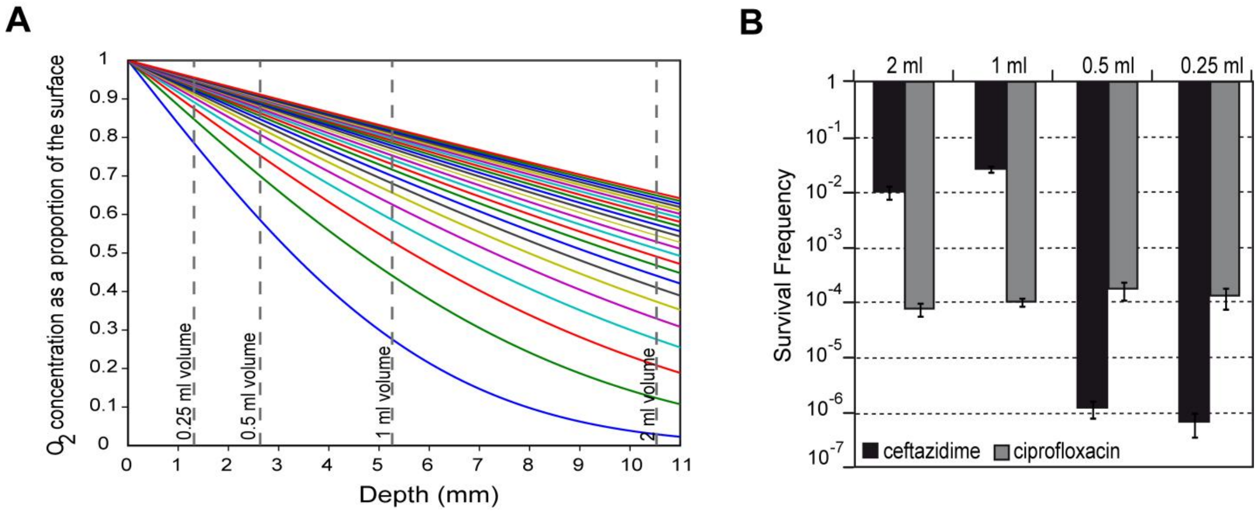


**Fig. S5. (A) One dimensional solution of $O_2$ diffusion into water.** It was assumed that the temperature of the water was 37 °C (diffusion coefficient of oxygen in water at this temperature is used) and that the initial oxygen content of the water was depleted to zero. The lowest line is the diffusion level after 1 hour and they progress upwards at one hour intervals for each line up to 24 hrs. The vertical lines represent the depth of medium in a 1.9 $cm^2$ (surface area of the base) cylindrical well plate at assay volumes of 0.25 ml, 0.5 ml, 1 ml and 2 ml. (**B)** The generation of an oxygen-limited bottom layer during static incubation was assessed by using different volumes of a mixture of bacteria and antibiotics and enumerating survival frequencies after 24 hrs incubation. The volumes correspond to a depth of 1.3 mm (0.25 ml), 2.6 mm (0.5 ml), 5.25 mm (1 ml), and 10.5 mm (2 ml) of the assay mixture inside the wells. In all experiments, bacteria grown to stationary phase were used and challenged with antibiotics under static incubation at 37 °C. Error bars represent the standard deviation over the mean from at least three independent experiments.

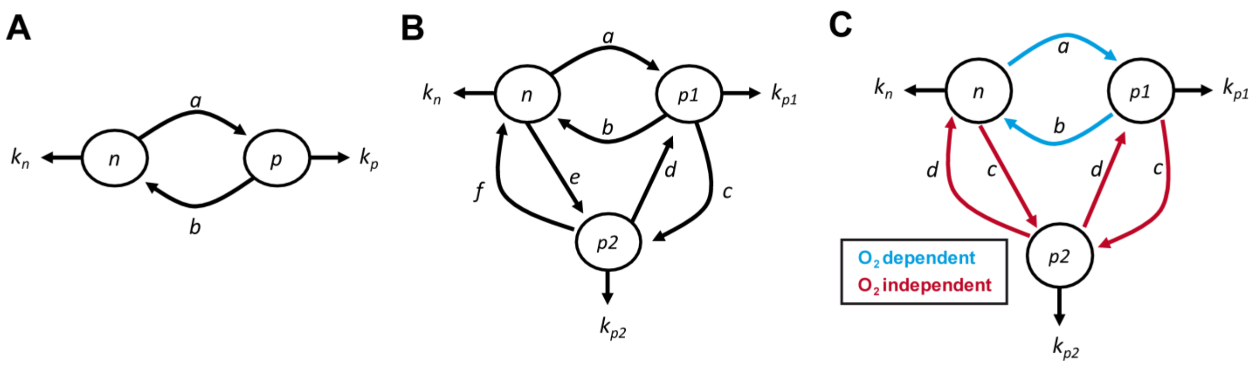


**Fig. S6. Two- and three-state switching models.** In all models presented, the different phenotypic population states are represented by circles and the switching rates between them by arrows. Under antibiotic treatment, a population state *i* declines (or grows if the drug is ineffective) at a rate $k_i$. **(A)** The original two-state stochastic switching model with a population of normal (*n*) and persister (*p*) cells and switching rates between them (*a* and *b*) (8). **(B)** The general three-state switching model with normal cells (*n*) and two distinct persister subtypes (*p1* and *p2*). **(C)** The modified three-state model with *p1* cells being tolerant to ceftazidime and *p2* cells being tolerant to ciprofloxacin. Blue arrows indicate that the switching rates between the normal susceptible cells *n* and *p1* (rates *a* and *b*) depend on the oxygen concentration. Red arrows indicate that the size of the ciprofloxacin-tolerant population *p2* is independent of the oxygen level. This is incorporated into the model by setting the switching rates $f = d$ and $e = c$.

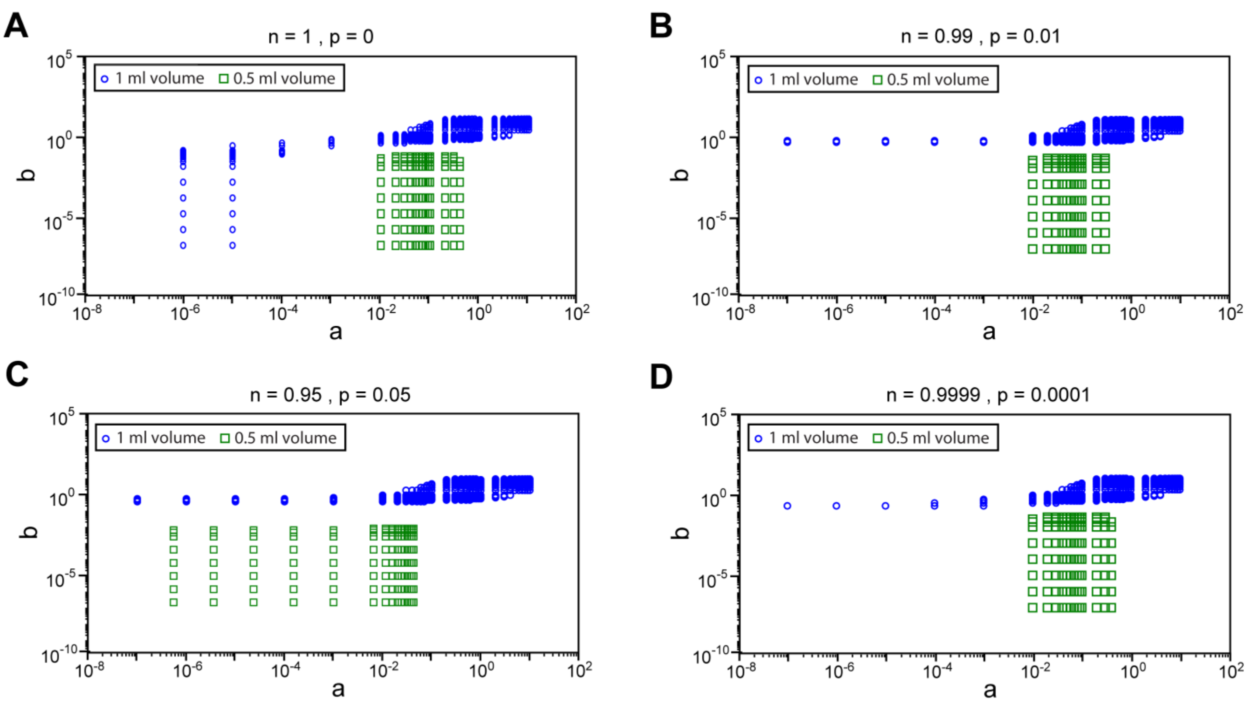


**Fig. S7. Parameter sweeps of the two-state model plotting only *a* and *b*.** In all plots, the green squares correspond to parameter sets that yield a low survivor number at 24 hrs of between 7.6 × $10^{-7}$ and 1.7 × $10^{-6}$, which corresponds to the survivor frequencies found after ceftazidime treatment in the 0.5 ml assay volume. The blue circles correspond to parameter sets that yield a high survivor number at 24 hrs and 48hrs of between 0.0238 and 0.0302 and 0.0106 and 0.037 respectively. These correspond to the survivor frequencies found after ceftazidime treatment in the 1 ml assay volume. **(A)** Initial population of $p$ = 0 and $n$ = 1. **(B)** Initial population of $p$ = 0.01 and $n$ = 0.99. **(C)** Initial population of $p$ = 0.05 and $n$ = 0.95. **(D)** Initial population of $p = 10^{-4}$ and $n = 1\text{-}10^{-4}$. Note that in all plots the two parameter sets are distinct and separate.

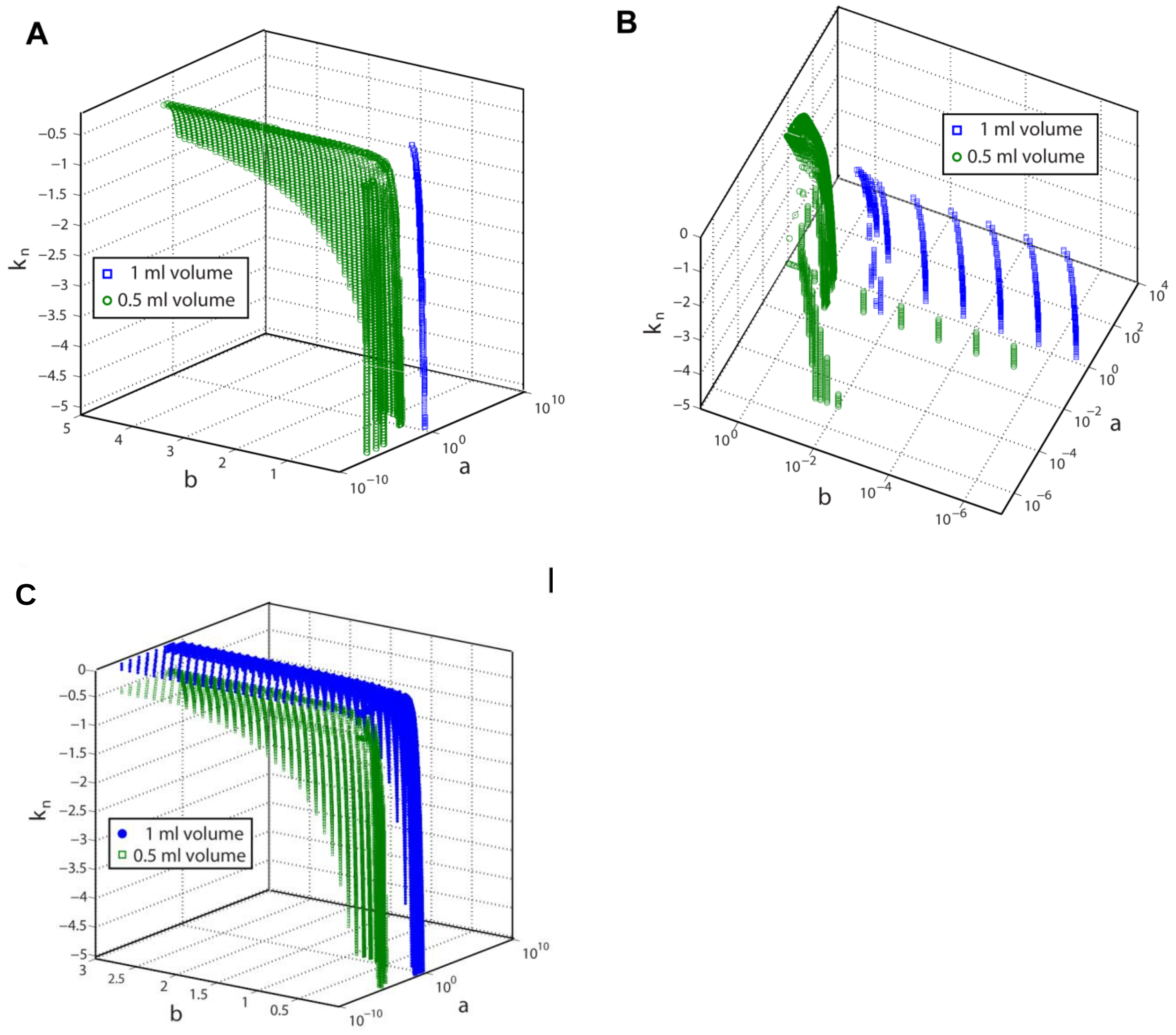


**Fig. S8. Extended parameter sweeps of the two-state model**. In both plots, each blue dot represents a distinct set of a, b and kn parameters of the two-state model (see Fig. S4A), which predict a survival frequency between $2.4 \times 10^{-2}$ and $3.0 \times 10^{-2}$ after 24 hrs. This corresponds to the survival frequencies observed after 24 hrs ceftazidime treatment in 1 ml volumes (see Fig. S2B). Every green square is a distinct set of a, b and kn parameters of the two-state model resulting in a survival frequency between 7.6x10-7 and 1.7x10-6 after 24 hrs. This corresponds to the survival frequencies observed after 24 hrs ceftazidime treatment in 0.5 ml volumes

(see Fig. S1B). **(A)** Parameter sweeps over ranges of $a$, $b$ and $k_n$ (assume $k_p = 0$), assuming an initial population of $p = 10^{-6}$ and $n = 1\text{-}10^{-6}$. **(B)** Parameter sweeps over ranges of $a$, $b$ and $k_n$ (assume $k_p = 0$), assuming an initial population of $p = 0$ and $n = 1$. **(C)** Solutions of the two-state model which conform to high and low survivor counts observed under different assay volumes *in vitro*. For these solutions, it is assumed that $k_p = 0$ and that at t = 0, $n = 1$ and $p = 0$. The parameter sweeps were run over the parameter ranges depicted on the axes.

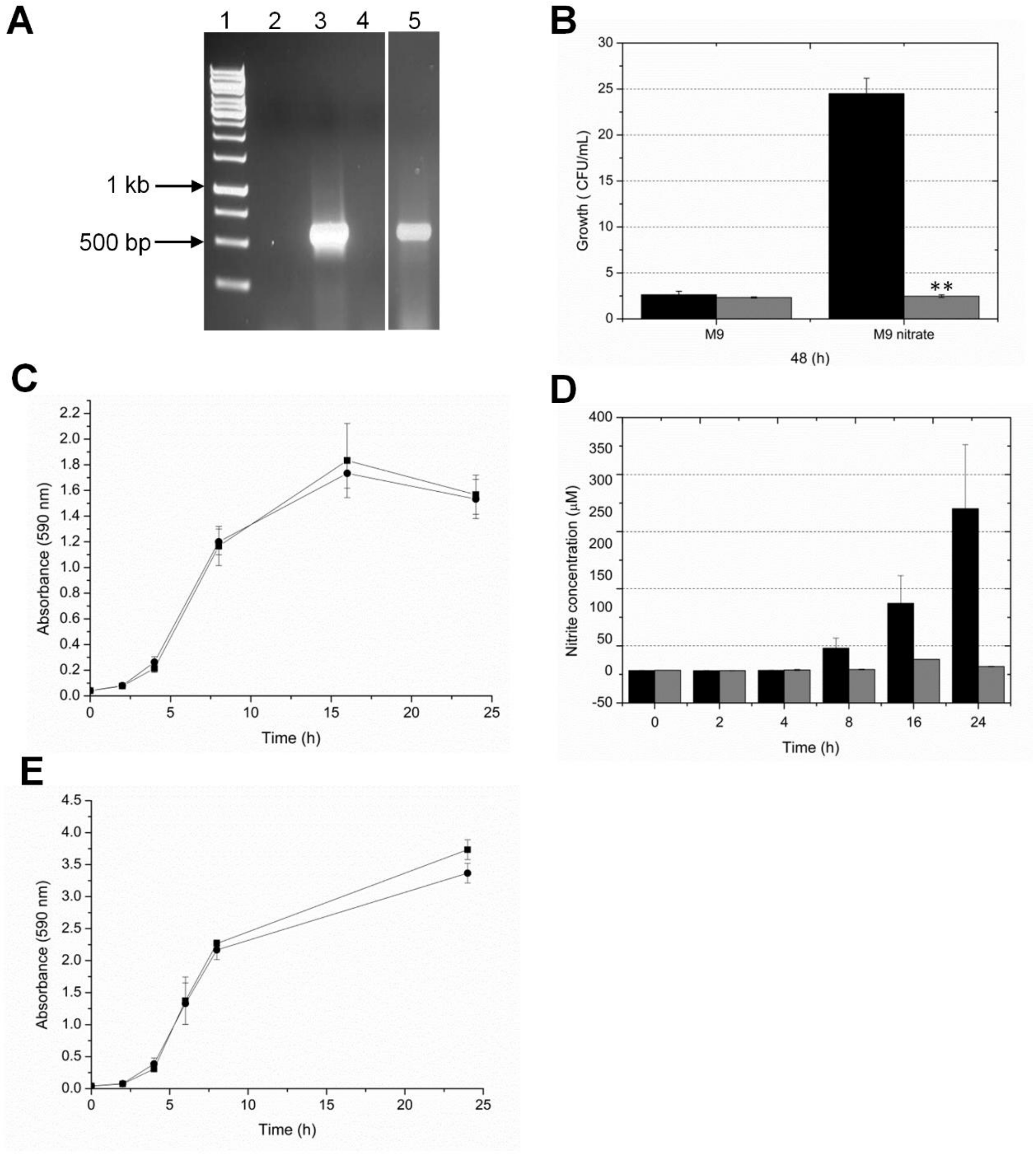


**Fig. S9. Deletion of *narG* in *B. pseudomallei* prevents anaerobic growth on nitrate and causes a significant reduction in nitrate reductase activity under aerobic conditions.** An in-frame nitrate reductase deletion mutant (Δ*narG*) was created using the suicide vector pDM4 carrying the BPSL2309 knockout cassette (pD2309). **(A)** PCR confirmation of the Δ*narG* deletion mutant using primers binding 300 bp up and downstream of the target gene (BPSL2309 **-** *narG*), generating a 600 bp product in the mutant and a much larger band in the wild-type (over 3,500 bp), not detected under the PCR conditions used. Lane 1 – 1 kb DNA ladder; lane 2 –

negative control ($H_2O$); lane 3 – pD2309; lane 4 – wild-type *B. pseudomallei* lysate; lane 5 – Δ*narG* colony lysate. Lack of a WT band and presence of 600 bp band for Δ*narG* confirms the deletion of BPSL2309. **(B)** Wild-type *B. pseudomallei* (black bars) and Δ*narG* (grey bars) were grown anaerobically in the presence or absence of 20 mM sodium nitrate. No anaerobic growth was seen for Δ*narG.* Data is the average of three independent biological replicates. Asterisks (**) denote statistically significant difference between WT and Δ*narG* ($P < 0.01$). **(C)** Aerobic growth in M9 minimal media supplemented with 20 mM sodium succinate and 20 mM sodium nitrate for wild-type (filled squares) and Δ*narG* (filled circles). Samples were taken throughout aerobic growth to determine the level of nitrate reductase activity in both *B. pseudomallei* cultures. **(D)** Relative nitrate reductase (NAR) activity was measured by determining the concentration of nitrite in the media using the Griess reagent system. Concentration of nitrite produced during aerobic growth was measured for the wild-type (black bars) or Δ*narG* (grey bars). The Δ*narG* deletion mutant could not reduce nitrate to nitrite, confirming a lack of NAR activity in this mutant. **(E)** Aerobic growth of wild-type *B. pseudomallei* and Δ*narG* in L-broth. Results presented are the mean of three biological replicates, each with two technical replicates used when performing the Griess reaction. Error bars ± standard deviation (SD).

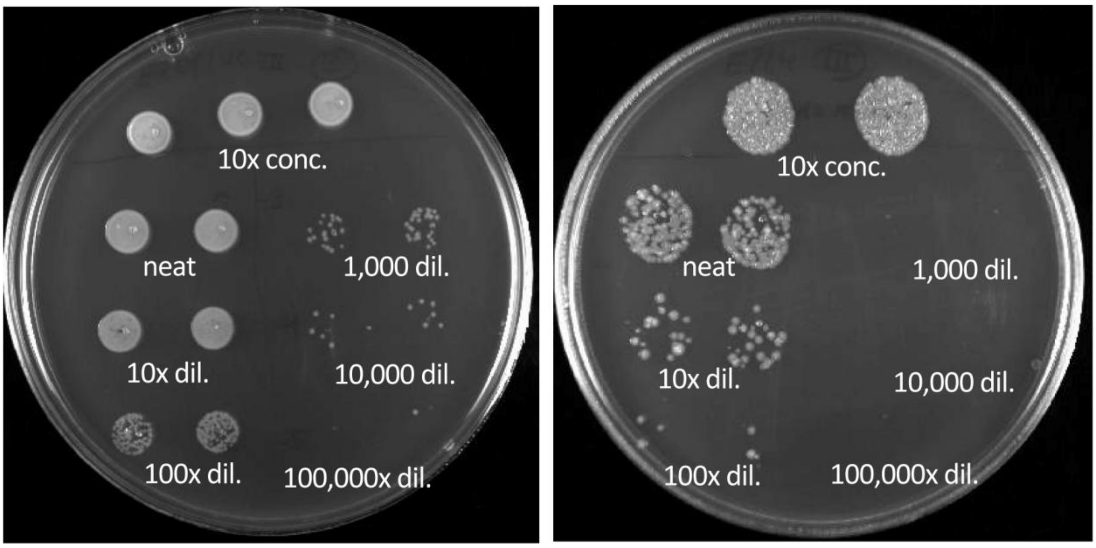


**Fig. S10. Phenotypes of different types of persister cells**. *B. thailandensis* strain E264 from stationary growth phase was challenged with 100 x MIC ceftazidime or 10 x MIC ciprofloxacin for 24 hours. Images represent LB agar plates with colonies derived from serial dilutions of washed surviving cells. The ceftazidime-derived persister plate shown in left panel was incubated for 24 hours at 37 °C, whereas the ciprofloxacin-derived plate shown in the right panel had been incubated for 48 hours at 37 °C.

**Table S1. Bacterial strains used in this study.**

| Strain | Description | Reference |
|---|---|---|
| *Burkholderia thailandensis* | | |
| E264 | Environmental isolate, sequenced strain | (19, 20) |
| DW503 | E264 efflux pump mutant (Δ*amrR*-*oprA*, $Km^S$ $Gm^S$ $Sm^S$) | (21) |
| Phuket 4W-1 | Water isolate from Thailand | (22) |
| CDC3015869 | Clinical isolate from Texas | (23) |
| CDC2721121 | Clinical isolate from Louisiana | (23) |
| *Burkholderia pseudomallei* | | |
| K96243 | Clinical isolate from Thailand, sequenced strain | (24) |
| K96243 Δ*narG* | Deletion of BPSL2309 in strain K96243 | This study |
| *Escherichia coli* | | |
| MG1655 | K-12 derivative ($F^-$ $\lambda^-$ *ilvG*$^-$ *rfb*-50 *rph*-1) | (25) |

**Table S2. Parameter sweeps of the two-state switching model.** The ranges shown are those that either conform to the high survivor numbers at 24 hrs and 48 hrs for the ceftazidime killing under 1 ml assay volume (High cefta) or the 24 hrs low survivor numbers at 0.5 ml assay volume (Low cefta) or the ciprofloxacin kill curve (Ciprofloxacin). Parameter ranges tested: $a \in (0, 10)$, $b \in (0, 10)$, $k_n \in (-5, 0)$, $k_p \in (-0.05, 0)$.

| Initial Conditions (n , p) | Tested Range Solution Ranges | Parameter ranges found to be within error ranges of high or low survivor numbers under ceftazidime treatment or to have agreed with ciprofloxacin kill curve (min, max) | | | |
|---|---|---|---|---|---|
| | | a | b | $k_n$ | $k_p$ |
| (1 , 0) | High ceftazidime | 0.002 , 0.04 | 0 , 0.05 | -0.5 , -.016 | -0.05 , 0 |
| | Low ceftazidime | $10^{-6}$ , 10 | $10^{-7}$ , 10 | -5 , -0.6 | -0.05 , 0 |
| | Ciprofloxacin | 0.02 , 0.03 | 0 , 0.12 | -2.2 , -1.7 | -0.1 , 0 |
| (0.99 , 0.01) | High ceftazidime | 0 , 0.04 | 0 , 0.05 | -0.5 , -0.17 | -0.05 , 0 |

| | | | | | |
|---|---|---|---|---|---|
| | Low ceftazidime | $10^{-7}$ , 10 | 0.04 , 10 | -5 , -0.6 | -0.05 , 0 |
| | Ciprofloxacin | 0.01 , 0.03 | 0.02 , 0.1 | -2.2 , -2 | -0.1 , -0.02 |
| (0.9999 , $10^{-4}$) | High ceftazidime | 0.01 , 0.4 | 0 , 0.04 | -5 , -0.25 | -0.05 , 0 |
| | Low ceftazidime | $10^{-7}$ , 10 | 0.02 , 10 | -5 , -0.6 | -0.05 , 0 |
| | Ciprofloxacin | None | None | None | None |
| (0.95 , 0.05) | High ceftazidime | 0 , 0.02 | 0 , 0.05 | -0.5 , -0.21 | -0.05 , 0 |
| | Low ceftazidime | $10^{-7}$ , 10 | 0.4, 10 | -5 , -0.6 | -0.05 , 0 |
| | Ciprofloxacin | None | None | None | None |

**Additional References**


1. **Hamad MA, Austin CR, Stewart AL, Higgins M, Vazquez-Torres A, Voskuil MI.** 2011. Adaptation and antibiotic tolerance of anaerobic *Burkholderia pseudomallei*. Antimicrob. Agents. Chemother. **55:**3313-3323.
2. **Andreae CA, Titball RW, Butler CS.** 2014. Influence of the molybdenum cofactor biosynthesis on anaerobic respiration, biofilm formation and motility in *Burkholderia thailandensis*. Res. Microbiol. **165:**41-49.
3. **Arena ME, Saguir FM, Manca de Nadra MC.** 1999. Arginine, citrulline and ornithine metabolism by lactic acid bacteria from wine. Int. J. Food Microbiol. **52:**155-161.
4. **Casiano-Colon A, Marquis RE.** 1988. Role of the arginine deiminase system in protecting oral bacteria and an enzymatic basis for acid tolerance. Appl. Environ. Microbiol. **54:**1318-1324.
5. **Chantratita N, Tandhavanant S, Wikraiphat C, Trunck LA, Rholl DA, Thanwisai A, Saiprom N, Limmathurotsakul D, Korbsrisate S, Day NP, Schweizer HP, Peacock SJ.** 2012. Proteomic analysis of colony morphology variants of *Burkholderia pseudomallei* defines a role for the arginine deiminase system in bacterial survival. J. Proteomics **75:**1031-1042.
6. **Keren I, Minami S, Rubin E, Lewis K.** 2011. Characterization and transcriptome analysis of *Mycobacterium tuberculosis* persisters. mBio **2**.
7. **Keren I, Shah D, Spoering A, Kaldalu N, Lewis K.** 2004. Specialized persister cells and the mechanism of multidrug tolerance in *Escherichia coli*. J. Bacteriol. **186:**8172-8180.

8. **Shah D, Zhang Z, Khodursky A, Kaldalu N, Kurg K, Lewis K.** 2006. Persisters: a distinct physiological state of *E. coli*. BMC Microbiol. **6:**53.

9. **Målen H, Berven FS, Fladmark KE, Wiker HG.** 2007. Comprehensive analysis of exported proteins from *Mycobacterium tuberculosis* H37Rv. Proteomics **7:**1702-1718.

10. **Hu Y, Movahedzadeh F, Stoker NG, Coates ARM.** 2006. Deletion of the *Mycobacterium tuberculosis* α-crystallin-Like *hspX* gene causes increased bacterial growth *in vivo*. Infect. Immun. **74:**861-868.

11. **Guisbert E, Yura T, Rhodius VA, Gross CA.** 2008. Convergence of molecular, modeling, and systems approaches for an understanding of the *Escherichia coli* heat shock response. Microbiol. Mol. Biol. Rev. **72:**545-554.

12. **Kiran M, Chauhan A, Dziedzic R, Maloney E, Mukherji SK, Madiraju M, Rajagopalan M.** 2009. *Mycobacterium tuberculosis ftsH* expression in response to stress and viability. Tuberculosis **89:**S70-S73.

13. **Fischer B, Rummel G, Aldridge P, Jenal U.** 2002. The FtsH protease is involved in development, stress response and heat shock control in *Caulobacter crescentus*. Mol. Microbiol. **44:**461-478.

14. **Rholl DA, Papp-Wallace KM, Tomaras AP, Vasil ML, Bonomo RA, Schweizer HP.** 2011. Molecular investigations of PenA-mediated beta-lactam resistance in *Burkholderia pseudomallei*. Front. Microbiol. **2**.

15. **Balaban NQ, Merrin J, Chait R, Kowalik L, Leibler S.** 2004. Bacterial persistence as a phenotypic switch. Science **305:**1622-1625.

16. **Richardson DJ.** 2000. Bacterial respiration: a flexible process for a changing environment. Microbiology **146:**551-571.

17. **Gonzalez PJ, Correia C, Moura I, Brondino CD, Moura JJ.** 2006. Bacterial nitrate reductases: Molecular and biological aspects of nitrate reduction. J. Inorg. Biochem. **100:**1015-1023.

18. **Tashiro Y, Kawata K, Taniuchi A, Kakinuma K, May T, Okabe S.** 2012. RelE-mediated dormancy is enhanced at high cell density in *Escherichia coli*. J. Bacteriol. **194:**1169-1176.

19. **Brett P, Deshazer D, Woods D.** 1998. *Burkholderia thailandensis* sp. nov., a *Burkholderia pseudomalle*i-like species. Int. J. Syst. Bacteriol. **48:**317-320.

20. **Yu Y, Kim HS, Chua HH, Lin CH, Sim SH, Lin D, Derr A, Engels R, DeShazer D, Birren B, Nierman WC, Tan P.** 2006. Genomic patterns of pathogen evolution revealed by comparison of *Burkholderia pseudomallei*, the causative agent of melioidosis, to avirulent *Burkholderia thailandensis*. BMC Microbiol. **6:**46.

21. **Burtnick M, Bolton A, Brett P, Watanabe D, Woods D.** 2001. Identification of the acid phosphatase (acpA) gene homologues in pathogenic and non-pathogenic *Burkholderia* spp. facilitates Tn*PhoA* mutagenesis. Microbiology **147:**111-120.

22. **Finkelstein RA, Atthasampunna P, Chulasamaya M.** 2000. *Pseudomonas* (*Burkholderia*) *pseudomallei* in Thailand, 1964–1967: geographic distribution of the organism, attempts to identify cases of active infection, and presence of antibody in representative sera. Am. J. Trop. Med. Hyg. **62:**232-239.

23. **Glass MB, Gee JE, Steigerwalt AG, Cavuoti D, Barton T, Hardy RD, Godoy D, Spratt BG, Clark TA, Wilkins PP.** 2006. Pneumonia and septicemia caused by *Burkholderia thailandensis* in the United States. J. Clin. Microbiol. **44:**4601-4604.

24. **Holden MTG, Titball RW, Peacock SJ, Cerdeno-Tarraga AM, Atkins T, Crossman LC, Pitt T, Churcher C, Mungall K, Bentley SD, Sebaihia M, Thomson NR, Bason N, Beacham IR, Brooks K, Brown KA, Brown NF, Challis GL, Cherevach I, Chillingworth T, Cronin A, Crossett B, Davis P, DeShazer D, Feltwell T, Fraser A, Hance Z, Hauser H, Holroyd S, Jagels K, Keith KE, Maddison M, Moule S, Price C, Quail MA, Rabbinowitsch E, Rutherford K, Sanders M, Simmonds M, Songsivilai S, Stevens K, Tumapa S, Vesaratchavest M, Whitehead S, Yeats C, Barrell BG, Oyston PCF, Parkhill J.** 2004. Genomic plasticity of the causative agent of melioidosis, *Burkholderia pseudomallei*. Proc. Natl. Acad. Sci. U.S.A. **101:**14240-14245.

25. **Guyer MS, Reed RR, Steitz JA, Low KB.** 1981. Identification of a sex-factor-affinity site in *E. coli* as gamma delta. Cold Spring Harb Symp Quant Biol **45 Pt 1:**135-140.
